# Comparative Efficacy of Seven Kinds of Chinese Medicine Injections in Acute Lung Injury and Acute Respiratory Distress Syndrome: A Network Meta-analysis of Randomized Controlled Trials

**DOI:** 10.3389/fphar.2021.627751

**Published:** 2021-03-09

**Authors:** Jie Guo, Jia Zhu, Qian Wang, Juan Wang, Yaodan Jia

**Affiliations:** ^1^ Department of Internal Medicine of TCM, The First Clinical Medical College, Jiangsu Province Hospital of Chinese Medicine, Affiliated Hospital of Nanjing University of Chinese Medicine, Nanjing, China; ^2^ Department of Chinese Medicine, Sir Run Run Hospital, Nanjing Medical University, Nanjing, China; ^3^ Department of Respiratory medicine, Jiangsu Province Hospital of Chinese Medicine, Affiliated Hospital of Nanjing University of Chinese Medicine, Nanjing, China; ^4^ Department of Intensive Medicine, Nanjing Hospital of Traditional Chinese Medicine Affiliated to Nanjing University of Chinese Medicine, Nanjing, China; ^5^ Environmental science, Faculty of Science, The University of Sydney, Sydney, NSW, Australia

**Keywords:** acute lung injury, acute respiratory distress syndrome, Chinese medicine injection, mortality, network meta-analysis, oxygenation index

## Abstract

**Background:** Chinese medicine injection is wildly used in Acute Lung Injury and Acute respiratory distress syndrome (ALI/ARDS) treatment. However, what kinds of CMIs are more effective in the ALI/ARDS treatment is uncertain.

**Objectives:** Compare the efficacy of different CMIs to identify the optimal one for the therapy of ALI/ARDS patients.

**Data sources:** We searched the data up to April 30, 2020 from MEDLINE, EMBASE, The Cochrane Library, Web of Science, the China Science Journal Citation Report (VIP database), WanFang and the China National Knowledge Infrastructure

**Study selection:** Randomized Clinical Trials assessed at least one of the following outcomes: mortality, Oxygenation Index, length of ICU stay, mechanical ventilation duration, APACHEⅡ score, SOFA score and Murray score, for adult patients of ALI/ADRS. Eligible Studies should also use CMIs as complementary therapies in addition to the standard treatment.

**Data extraction and synthesis**: Two reviewers independently assessed the data. Then, we used a Bayesian random-effects network meta-analysis for data synthesis.

**Results:** Twenty-six studies were selected (involved 2073 participants). Seven kinds of CMIs were evaluated. Compared with standard treatment, Xuebijing is associated with lower mortality. Tanreqing and Xuebijing have the best effect on improving the Oxygenation Index. Huangqi, Danshen, Tanreqing and Xuebijing can significantly reduce the APACHE II score (Huangqi works better than Xuebijing). Huangqi and Xuebijing have the best effect on reducing mechanical ventilation duration and Murray score, while Xuebijing has the best effect on shortening the length of ICU stay.

**Conclusions:** As adjuvant drugs, Xuebijing, Tanreqing and Huangqi show certain effects on treating ALI/ARDS in different aspects.

## Introduction

Acute Lung Injury/Acute respiratory distress syndrome (ALI/ARDS) is one of the main causes of death in the critical care unit which characterized by progressive hypoxemia and respiratory distress. It is caused by severe infection, shock, trauma and other non-cardiogenic pathogenic factors, resulting in a sharp decline in pulmonary gas exchange function. However, relatively few treatments are available in treating ALI/ARDS. In addition to controlling the primary disease and providing respiratory support, researchers hope to find drugs that work on the pathological mechanism (including alveolar flooding and pulmonary edema formation, cytokine storm, coagulation and fibrinolysis disorders, etc.), such as β2 agonists, statins, keratinocyte growth factor, glucocorticoid, alveolar surfactant, antioxidant, even cytokine monoclonal antibodies and antagonists. Unfortunately, no pharmacologic treatments aimed at the underlying pathology have been shown to be effective or been recommended by the guideline (Chinese Medical Association, 2006; Zhang et al., 2008; Fan et al., 2018). More adjuvant treatments are still being studied in order to reduce the mortality of ALI/ARDS. In China, Traditional Chinese Medicine, as a complementary therapy, has played a certain role in the treatment of ALI/ARDS patients (Chen et al., 2019). In particular, since the epidemic of COVID-19, Traditional Chinese Medicine has been reported to reduce the conversion rate of severe cases (Luo et al., 2020), and shown a potential effect in treating ALI/ARDS patients caused by severe COVID-19 (Chan et al., 2020).

Traditional Chinese medicine and treatment includes Chinese herbal decoction, acupuncture and Chinese medicine injection (CMI). Compare with the other two methods, CMI has a more-clear composition and is easier to standardize. Therefore, it is worth popularizing if CMI proved to be effective in the treatment of ALI/ARDS. Many kinds of CMIs are being used in clinical treatment, such as Xuebijing, Tanreqing and Shenmai. Some clinical trials showed that CMIs are effective for treating ALI/ARDS, but there is no study to compare the efficacy of these drugs. In clinical, CMIs are usually selected based on the method of "treatment based on syndrome differentiation", but this is only applicable to Chinese medicine doctors. For most physicians, they cannot choose drugs based on TCM theory. Therefore, we compare the efficacy of different CMI through the method of Bayesian network meta-analysis, hoping to find one or more CMIs that may be more effective as a reference for clinicians.

This article followed the steps outlined by the Cochrane Collaboration (Higgins JPT) and the Preferred Reporting Items for Systematic Reviews and Meta-analyses (PRISMA) reporting guidelines (Hutton et al., 2015), and also refer to the PRISMA-CHM 2020 (Zhang et al., 2020).

## Methods

First of all, we pre-searched the articles published by Chinese scholars on the treatment of ALI/ARDS with CMIs, and preliminarily identified several kinds of CMIs that are widely used to treat ALI/ARDS in clinic. Then we worked out the research plan according to the advice of experts. This study has registered on PROSPERO (No.: CRD42020181369).

### Eligibility Criteria

1) Randomized Clinical Trials. 2) Adult patients (older than 18 years old) who were diagnosed with ALI/ARDS defined by American-European Consensus Conference (AECC) in 1994 and Berlin definition (2012) were included. Since most of the Chinese literature uses the diagnostic criteria of the Chinese Medical Association, we confirmed that the diagnostic criteria and treatment standard of CMA in 2000 and 2006 followed those of AECC in 1994, so the literatures using these two diagnostic criteria were also be included in our study (Bernard et al., 1994; Chinese Medical Association, 2000; Chinese Medical Association, 2006; Ranieri et al., 2012). 3) All participants received standard treatment for ALI/ARDS according to the guidelines. Experimental groups use CMIs as complementary therapies in addition to the standard treatment. 4) Primary outcomes include mortality and Oxygenation Index (PaO2/FiO2, we only recorded the changes of Oxygenation Index before and after seven days of treatment if studies reported Oxygenation Index at different time points). Secondary outcomes include the length of ICU stay, mechanical ventilation duration, APACHEⅡscore, SOFA and Murray score. 5) Articles that are repeatedly published or with incomplete data or do not have access to the full text would be eliminated. If several articles come from the same study, only the one with the largest number of participants and the latest data was included.

### Literature Search and Study Selection

We performed an electronic search of MEDLINE, EMBASE, The Cochrane Library, Web of Science, the China Science Journal Citation Report (VIP database), WanFang and the China National Knowledge Infrastructure (CNKI) without any language restrictions. All the publications until April 30, 2020 were searched. Search terms and relative variants include Traditional Chinese Medicine, injections, xuebijing, tanreqing, reduning, xiyanping, qingkailing, shengmai, shenmai, zhenqifuzheng, shenqifuzheng, chuanxiongqin, shenfu, danshen, yanhuning, acute respiratory distress syndrome, acute lung injury, acute respiratory failure. Additionally, the reference lists of key articles (such as reviews, multi-center researches) were also screened for seeking the potentially relevant articles (Search strategy in Supplementary Data).

Firstly, two review authors (Guo J and Wang J) independently screened the titles and abstracts after removing duplicate records. Then the full texts of these studies were assessed independently for eligibility. The disagreements in between were resolved through discussion with the third author (Wang Q).

### Data Extraction and Risk-of-Bias Assessment

We use standardized pre-piloted tables to extract data from included studies for assessment. The Cochrane Risk of Bias Tool (Higgins JPT et al., 2019) was used to evaluate the quality of these studies. Two review authors (Guo J and Wang J) independently assessed the risk of bias in included studies by considering random sequence generation, allocation concealment, blinding implementation, incomplete outcome data and selective reporting. The differences among the authors on the risk of bias would be resolved through discussion with the third author (Wang Q) where necessary. Funnel plots were used to assess publication bias if an outcome has more than 10 trials in each arm (Higgins JPT et al., 2019).

We conducted the heterogeneity test by L’Abbe graph and calculate I^2^, and attempted to explain the heterogeneity through sensitivity analyses and meta-regression. Characteristics of the studies such as age, dosage, treatment period, etc. would be displayed (shown in Table 1) to help identify potential clinical reasons for heterogeneity. As all studies are two-arm design, assessment of consistency was not needed.

**TABLE 1:** characteristics of included studies.

Source	Study period	Total (experimental/control) no. of patients	Experimental/control age, mean (SD), y	Intervention/daily dosage	Treatment period, day	Outcomes^a^
Qian et al. (2010)	2007.1–2009.6	59 (29/30)	78.5(5.8)/77.9(6.9)	Reduning/20 ml	7	①②
Chen and Li (2011)	2006.1–2009.12	60 (30/30)	64 (35)	Xuebijing/100 ml	7	①
Liang and Peng (2011)	2008.5–2010.5	56 (28/28)	57.2(10.8)/58.3(10)	Xuebijing/200 ml	7	①③
Cao (2012)	2010.9–2011.12	80 (40/40)	65.7/66.3	Shenmai/50 ml	10	②④⑥
Jiang et al. (2012)	2008.8–2010.8	59 (29/30)	42(13)/43(11)	Xuebijing/150 ml	7	②⑤
Liang (2012)	2010.11–2011.4	70 (35/35)	39.6 (8.3)	Xuebijing/200 ml	7	②
Liu et al. (2012)	2008.1–2010.7	172 (91/81)	55/53	Xuebijing/200 ml	7	①②③④⑤⑥
Ma (2012)	2009.1–2012.1	60 (30/30)	57.5(8.3)/57.2(8.7)	Xuebijing/100 ml	7	②③④⑤
Tian and Sun (2012)	2010.1–2011.1	100 (50/50)	39.5 (3.7)	Xuebijing/100 ml	7	①③④
Zhang et al. (2012)	2009.11–2011.9	60 (30/30)	66.9 (17.3)	Danshen/30 ml	5	①②③⑤
Chen et al. (2013)	—	100 (50/50)	50(15.1)/52.4(15.9)	Danshen/30 ml	7	②
Li et al. (2013)	2011.6–2013.1	60 (30)/(30)	46.2(17.9)/40.3(15.3)	Huangqi/20 ml	7	①④⑤⑥
Li (2013)	2012.5–2013.4	40 (20/20)	65.2(12.6)/66.6(10.1)	Tanreqing/20 ml	7	⑤⑥
Yang (2013)	2010.3–2012.4	124 (62/62)	44.1(7.5)/43.5(6.7)	Tanreqing/20 ml	7	①②⑤
Zhang (2014)	2009.1–2014.2	47 (23/24)	44(7.5)/43.6(6.7)	Tanreqing/20 ml	7	②
Wang et al. (2015)	2014.1–2014.10	100 (50/50)	42.2(4.1)/43.4(4.6)	Huangqi/20 ml	5	③④
He (2016)	2015.1–2016.4	84 (42/42)	58(9)/59(10)	Xuebijing/100 ml	7	②⑤
Lin et al. (2016)	2013.6–2015.7	80 (40/40)	48.7 (8.8)	Fufangkushen/30 ml	-	①②④
Liu et al. (2016)	2013.1–2016.4	60 (30/30)	54.3(5.9)/53.8(6.2)	Xuebijing/100 ml/150 ml	14	②③④
Deng (2017)	2015.5–2016.5	70 (35/35)	42(6)/42(7)	Xuebijing/150 ml	7	②⑤
Gou et al. (2017)	2012.8–2014.10	40 (20/20)	40.6/45.2	Xuebijing/200 ml	7	②
Guo et al. (2018)	2013.12–2017.12	136 (68/68)	51.1(11.1)/49(9.2)	Tanreqing/20 ml	7	②
Wang (2018)	—	132 (66/66)	62.6(3.2)/62.5(2.4)	Xuebijing/100 ml	7	⑤⑥
Zhen et al. (2019)	2016.6–2018.6	64 (32/32)	53.4(6.2)/52.3(7.7)	Xuebijing/100 ml	7	②③④⑤
Chang et al. (2019)	2016.1–2019.1	60 (30/30)	51.3(7.2)/53.6(7.9)	Xuebijing/100 ml	7	①②
Lang and Lei, (2019)	2017.7–2018.8	100 (50/50)	47.3(3.4)/46.4(3.5)	Xuebijing/150 ml	7	②

^a^Outcome ① mortality; ② PaO_2_/FiO_2_ in 7 days after treatment; ③ length of ICU stay; ④ duration of mechanical ventilation; ⑤ APACHEII score; ⑥ Murray score.

### Statistical Analysis

We determined risk ratios (RR, for dichotomous outcomes) or mean difference (MD, for continuous outcomes) and as well as 95% credible intervals (Gattinoni et al., 2001) by the Bayesian hierarchical random-effects model to get the effect sizes (Warn et al., 2002). A difference was considered statistically significant when the range of the 95% CrI of RR did not include one, or the range of the 95% CrI of MD did not include zero.

The calculation was achieved by using the Markov chain Monte Carlo simulation with noninformative prior distributions (Bodnar et al., 2017). The model was run with four chains and 50,000 iterated simulations, discarding the initial 20,000 iterations as burn-in. We assessed model fit by residual deviance, leverage, and the deviance information criterion (Hutton et al., 2015). Smaller Dbar value indicates a better model fit; pD denotes the effective number of parameters (leverage); lower DIC value indicates a better model fit (Spiegelhalter et al., 2002). In addition, the convergence was evaluated by the potential scale reduction parameter (PSRF) of each group, if each PSRF is close to 1, the convergence is good.

For all the interventions, we made a pairwise comparison of the effect as well as plotting a surface under cumulative ranking (SUCRA) curve to evaluate the superior or inferior of them. Stata12.0 software and GeMTC package of R software were used to realize the statistical process.

## Results

**FIGURE 1:**
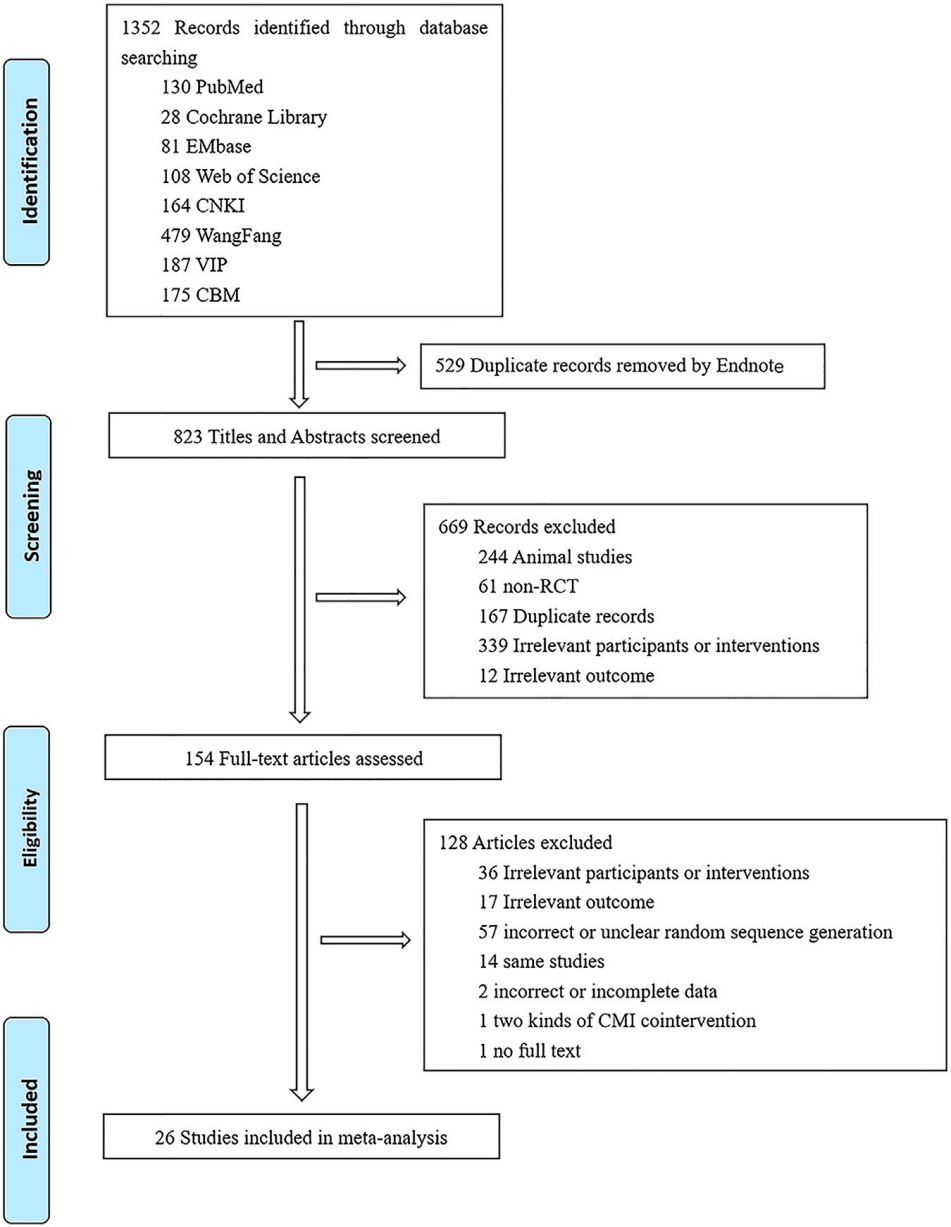
Flow Diagram of literature screening.

We identified 1,352 records through database searching. After removing the duplicate records, screening by titles and abstracts, and assessing full text, twenty-six studies (Qian et al., 2010; Chen and Li, 2011; Liang and Peng, 2011; Cao, 2012; Jiang et al., 2012; Liang, 2012; Liu et al., 2012; Ma, 2012; Tian and Sun, 2012; Zhang et al., 2012; Chen et al., 2013; Li, 2013; Li et al., 2013; Yang, 2013; Zhang, 2014; Wang et al., 2015; He, 2016; Lin et al., 2016; Liu et al., 2016; Deng, 2017; Gou et al., 2017; Guo et al., 2018; Wang, 2018; Chang et al., 2019; Lang and Lei, 2019; Zhen et al., 2019) were included in our final study (shown in Figure 1), which involved in 2073 participants. Seven kinds of CMIs including Xuebijing, Tanreqing, Danshen, Huangqi, Reduning, Shenmai and Fufangkushen were selected (detailed information were shown in Table 2). Among the referenced studies, Xuebijing was used in fifteen studies, Tanreqing was used in four studies, Danshen was used in two studies, Huangqi was used in two studies, Reduning, Shenmai and Fufangkushen were used in one study respectively.

**TABLE 2:** Detailed information of Seven kinds of CMIs.

Name	Source	Species, concentration	Indications^a^	Quality control reported? (Y/N)	Chemical analysis reported? (Y/N)
Reduning	*Gardenia jasminoides J.Ellis* (Fructus Gardeniae), measured as glycosides (C17H24O10) 180-280 mg, *Lonicera japonica* Thunb., measured as chlorogenic (C16H18O9) acid 112-168 mg, *Artemisia annua L.*, the content is not mentioned in the pharmacopeia	Fructus Gardeniae, measured as glycosides (C17H24O10) 180-280 mg, Lonicera japonica Thunb., measured as chlorogenic (C16H18O ) acid 112mg-168 mg, *Artemisia annua,* the content is not mentioned in the pharmacopeia	① Infectious diseases include: Common cold, acute tracheitis and bronchitis, community acquired pneumonia, asthmatic bronchitis; ② Virus infection include: Human infection with H7N9 avian influenza, severe fever with thrombocytopenia syndrome, influenza A (H1N1), dengue fever, COVID-19; ③ Critically ill include: Sepsis, severe and critical COVID-19	Y- national food and drug administration national drug standards. Standard number: YBZ08202005-2015Z	Y- HPLC and GC
Xuebjing	*Carthamus tinctorius L.* (Carthami Flos), *Paeonia lactiflora Pall.* (Paeoniae Radix Rubra), *Conioselinum tenuissimum* (Nakai) Pimenov and Kljuykov (syn. Ligusticum tenuissimum Kitag.), *Conioselinum anthriscoides* (H.Boissieu) Pimenov and Kljuykov (syn. Ligusticum sinense Oliv.), and *Conioselinum smithii* (H.Wolff) Pimenov and Kljuykov (syn. Ligusticum jeholense Nakai et Kitag.) (Ligusticum Rhizoma),. *Salvia miltiorrhiza Bunge* (Salvia miltiorrhiza Radix), *Angelica sinensis* (Oliv.) Diels (Angelicae Sinensis Radix).	*Carthami Flos*, *Paeoniae Radix Rubra*, *Ligusticum Rhizoma*, *Salvia miltiorrhiza Radix*, *Angelicae Sinensis Radix*	① Infectious diseases include: Community acquired pneumonia ② Virus infection include: Human infection with H7N9 avian influenza, severe fever with thrombocytopenia syndrome, dengue fever, COVID-19 ③ Critically ill include: Multiple organ dysfunction syndrome in the elderly, stroke -associated pneumonia, acute pancreatitis, acute paraquat intoxication, severe and critical COVID-19, sepsis, sepsis complicated with ALI, sepsis complicated with disseminated intravascular coagulation, ALI/ARDS ④ Others: Chronic pulmonary heart disease	Y- national food and drug administration national drug standards. Standard number: YBZ01242004-2010Z-2012	N
Shenmai	*Panax ginseng* C. A. Mey. (Red Ginseng), measured as ginsenoside (C48H82O18) 40 mg. Ophiopogon japonicus (Thunb.) Ker Gawl. (Radix Ophiopogonis), the content is not mentioned in the pharmacopeia	*Red Ginseng*, measured as ginsenoside (C48H82O18) 40 mg, Radix Ophiopogonis, the content is not mentioned in the pharmacopeia	① Infectious diseases include: Community acquired pneumonia ② Virus infection include: Human infection with H7N9 avian influenza, severe fever with thrombocytopenia syndrome, dengue fever, influenza A (H1N1), COVID-19 ③ Critically ill include: Acute ischemic stroke, acute myocardial infarction, sepsis, severe and critical COVID-19 ④Others: Chronic heart failure	Y- national food and drug administration national drug standards. Standard number: WS3-B-3428-98-2010Z	Y-HPLC
Danshen	*Salvia miltiorrhiza Bunge* (Salvia miltiorrhiza Radix) 45 g, content 6 mg protocatechualdehyde (C7H6O3) at least	*Salvia miltiorrhiza* Radix 45 g, content 6 mg protocatechualdehyde (C7H6O3) at least	① virus infection include: Influenza A (H1N1) ② Critically ill include: Stroke, acute myocardial infarction, sepsis, acute pancreatitis ③ Others: Chronic pulmonary heart disease, allergic purpura disease, chronic glomerulonephritis, chronic cerebral ischemia	Y- national food and drug administration national drug standards. Standard number: WS3-B-3766-98-2011	Y-HPLC
Huangqi	*Astragalus mongholicus Bunge* (syn. Astragalus membranaceus) 40 g, content 1.6 mg Astragalus Saponin I (C41H68O14) at least	*Astragalus membranaceus* 40 g, content 1.6 mg Astragalus Saponin I (C41H68O14) at least	① Virus infection include: Chronic hepatitis B ② Critically ill include: Acute myocardial infarction, sepsis ④ Others: Chronic heart failure, chronic glomerulonephritis	Y- national food and drug administration national drug standards. Standard number: WS3-B-3335-98	Y-TLC
Tanreqing	Scutellaria baicalensis Georgi, content 100 mg baicalin (C21H18O11) at least, Fel Ursi, (content 108 mg ursodeoxycholic acid (C24H40O4) at least,. Naemorhedus goral Hardwicke, Lonicera japonica Thunb. Forsythia suspensa (Thunb.) Vahl, the content is not mentioned in the pharmacopeia	*Scutellaria baicalensis* Georgi, content 100 mg baicalin (C21H18O11) at least, Fel Ursi, (content 108 mg ursodeoxycholic acid (C24H40O4) at least, Naemorhedus goral Hardwicke, Lonicera japonica Thunb. Forsythia suspensa, the content is not mentioned in the pharmacopeia	① Infectious diseases include: Common cold, acute tracheitis and bronchitis, community acquired pneumonia ② Virus infection include: Human infection with H7N9 avian influenza, dengue fever, COVID-19 ③ Critically ill include: Sepsis, severe and critical COVID-19 ④ Others: Chronic pulmonary heart disease, chronic obstructive pulmonary disease	Y- national food and drug administration national drug standards. Standard number: YBZ00912003-2007Z-2009-2012	Y-HPLC
Fufangkushen	Sophora flavescens Aiton (Radix Sophorae Flavescentis), 42 g, content 540 mg matrine (C16H24N2O) at least, Smilax bockii Warb. (syn. Heterosmilax japonica Kunth),18 g	*Radix Sophorae Flavescentis*, 42 g, content 540 mg matrine (C16H24N2O) at least, Heterosmilax japonica Kunth,18 g	Complementary treatment of tumors	Y- national food and drug administration national drug standards. Standard number: WS3-B-2752-97-2014	Y- TLC

^a^These indications are mentioned in the clinical guidelines listed in Supplementary Data.

### Risk of Bias

**FIGURE 2:**
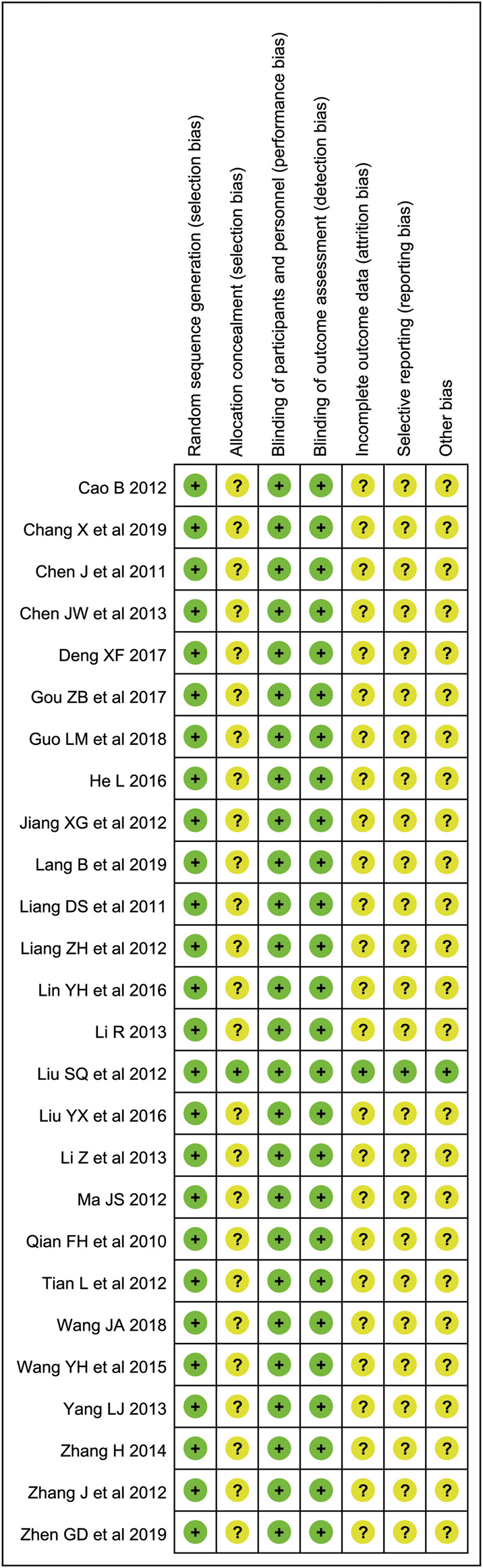
Evaluation of literature quality: The green dot represents low risk; the yellow dot represents uncertain risk; the red dot represents high risk.

The random sequence generation of each study was rated as low risk as they all described the randomization with an explicit random method. Only one piece of literature described the allocation concealment (sealed envelopes). We evaluated the performance bias and detection bias as low risk of each study, although only two studies were blind (one was single-blind and the other was double-blind). Because the outcomes are objective, the scores were all calculated by objective indicators, both patients and researchers were little affected by blinding. Attrition bias and reporting bias of one article was rated as low risk, the others were uncertain (shown in Figure 2).

### Heterogeneity

We analyzed the heterogeneity for each outcome. The L’Abbe diagram of mortality showed no heterogeneity (Supplementary Figure 1). Forest Plots of other outcomes showed moderate or high heterogeneity (Supplementary Figure 2). After using the random-effects model, the heterogeneity decreased to a low level in each outcome (Supplementary Table 1).

### Mortality

**FIGURE 3:**
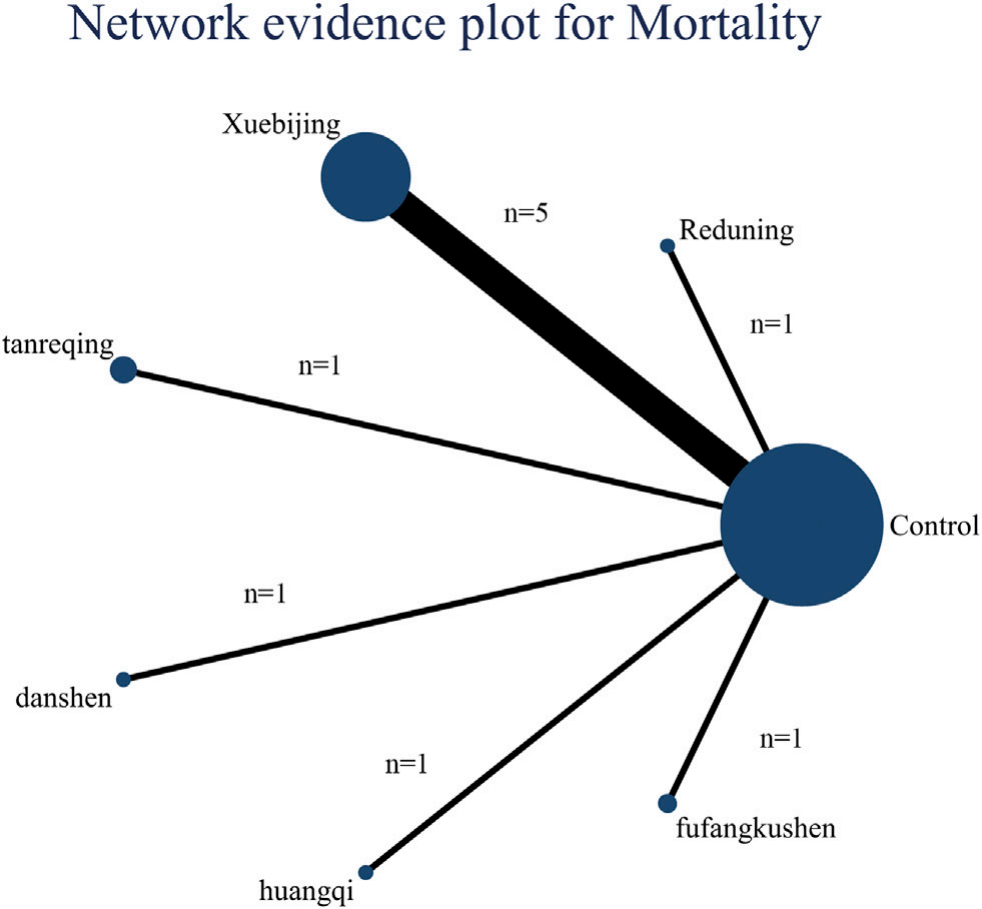
Network evidence plot for mortality: The nodes represent interventions. Larger nodes indicate larger total number of participants in arms. The connections between nodes represent direct comparisons. The width of the connection line is proportional to the number of trials.

**FIGURE 4:**
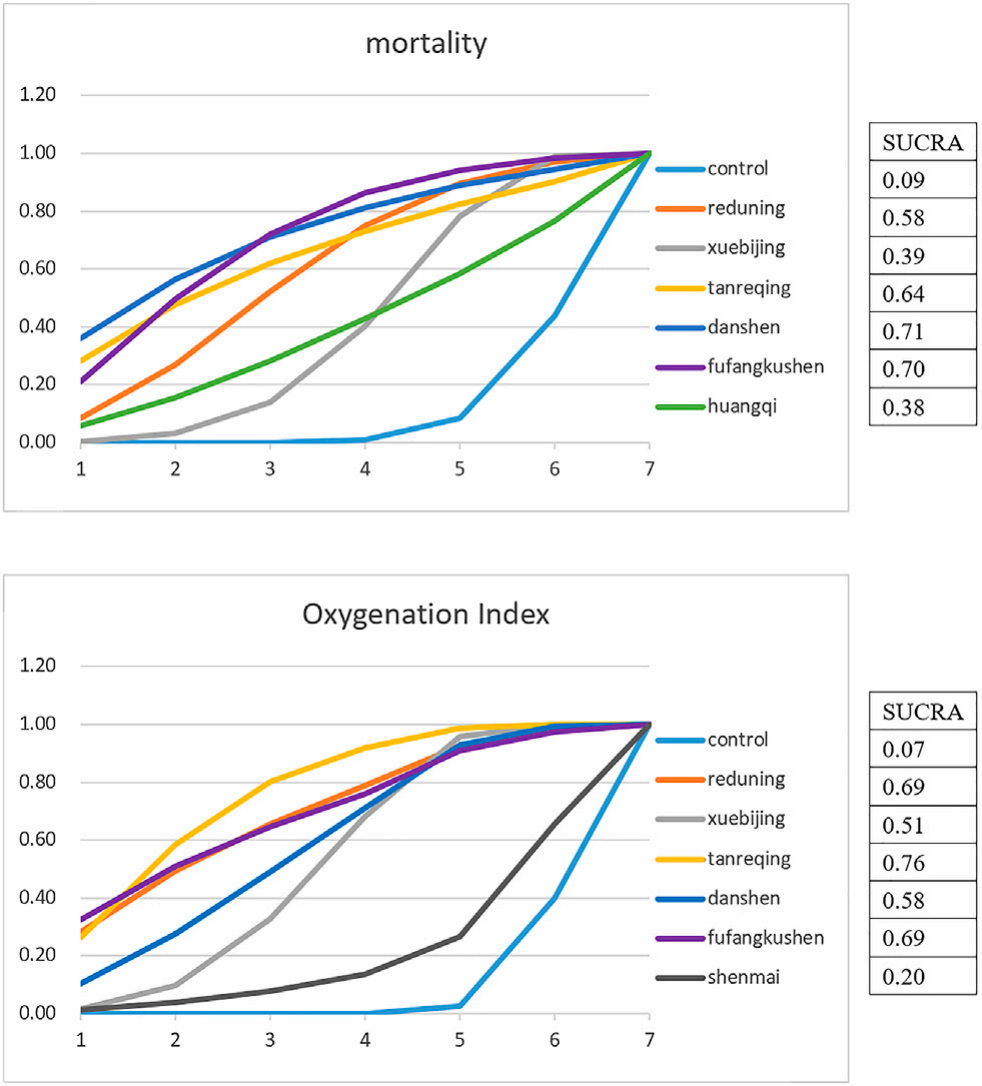
Cumulative probabilities for the Effect of Interventions on the primary outcomes: Each line represents an intervention. The surface area under the curve is constructed according to the ranking probability of all interventions. The *x*-axis represents the ranking of the strategy, and the *y*-axis represents the probability of each rank. The larger the area, the greater the probability and optimality.

Ten articles reported the mortality, including six interventions, the evidence plot is shown in Figure 3. Rank for the efficacy of six kinds of CMIs based on SUCRA are as follows: Danshen (SUCRA = 0.71), Fufangkushen (SUCRA = 0.70), Tanreqing (SUCRA = 0.64), Reduning (SUCRA = 0.58), Xuebijing (SUCRA = 0.39), Huangqi (SUCRA = 0.38) (shown in Figure 4).

However, the results of the pairwise comparison (Table 3) showed that only Xuebijing had a statistical difference compared with the control group (RR = 0.63, 95%CrI:0.42, 0.94). No statistical difference was found among the six kinds of CMIs. The distribution was roughly symmetrical in funnel plots, which indicated that there was no publication bias and small sample effect (Supplementary Figure 3A).

**TABLE 3:** Comparison of the included CMIs in Risk Ratio (95% CrI) for mortality.

Control	0.46 (0.17, 1.12)	0.63 (0.42, 0.94)	0.36 (0.04, 1.97)	0.31 (0.04, 1.43)	0.67 (0.16, 2.46)	0.35 (0.11, 1.01)
2.18 (0.89, 5.95)	Reduning	1.39 (0.51, 3.98)	0.79 (0.08, 5.68)	0.67 (0.07, 4.28)	1.46 (0.26, 7.53)	0.77 (0.17, 3.3)
1.58 (1.06, 2.41)	0.72 (0.25, 1.96)	Xuebijing	0.57 (0.07, 3.27)	0.48 (0.06, 2.45)	1.05 (0.24, 4.19)	0.56 (0.15, 1.73)
2.77 (0.51, 22.58)	1.26 (0.18, 12.28)	1.76 (0.31, 14.88)	Tanreqing	0.84 (0.06, 10.91)	1.82 (0.2, 22.49)	0.96 (0.13, 10.44)
3.25 (0.7, 27.45)	1.5 (0.23, 15.09)	2.07 (0.41, 17.76)	1.2 (0.09, 15.98)	Danshen	2.23 (0.26, 25.78)	1.15 (0.16, 12.14)
1.5 (0.41, 6.31)	0.68 (0.13, 3.79)	0.95 (0.24, 4.17)	0.55 (0.04, 5.03)	0.45 (0.04, 3.92)	Huangqi	0.52 (0.09, 3.08)
2.85 (0.99, 9.49)	1.3 (0.3, 5.77)	1.8 (0.58, 6.45)	1.05 (0.1, 7.88)	0.87 (0.08, 6.12)	1.92 (0.32, 11.02)	Fufangkushen

### Oxygenation Index

**FIGURE 5:**
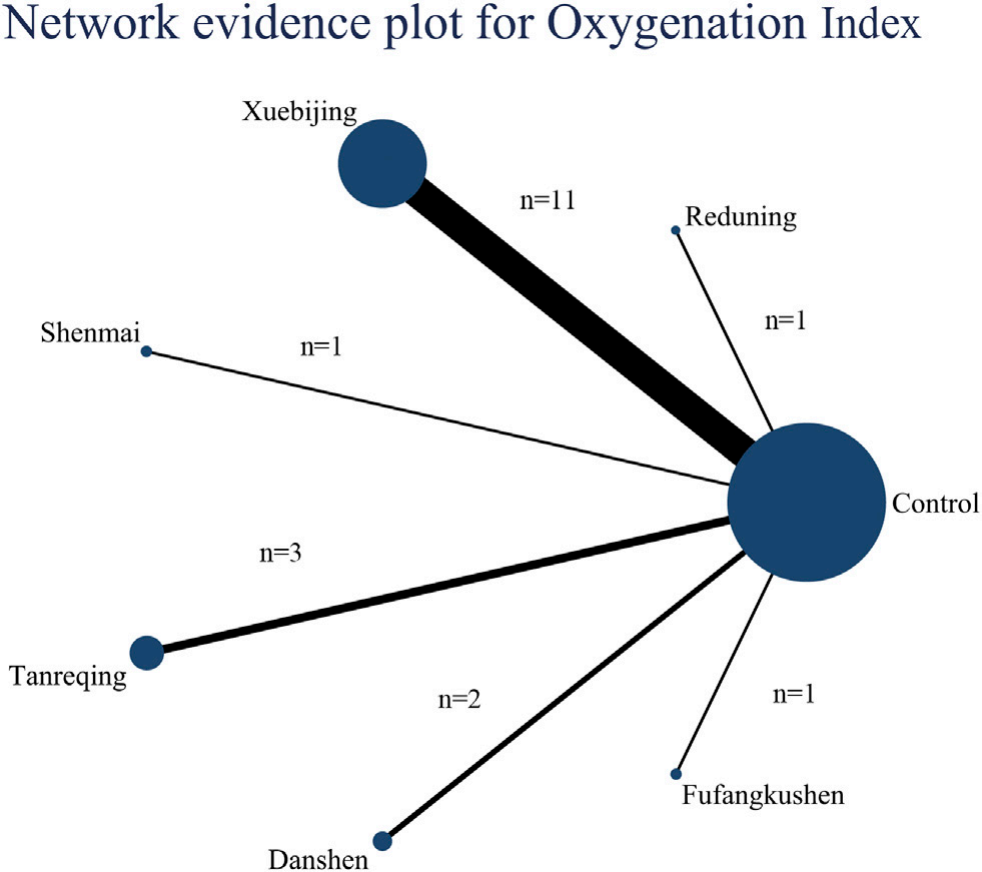
Network evidence plot for Oxygenation Index: The nodes represent interventions. Larger nodes indicate larger total number of participants in arms. The connections between nodes represent direct comparisons. The width of the connection line is proportional to the number of trials.

Nineteen articles reported the variation of Oxygenation Index after seven days’ treatment, the evidence plot is shown in Figure 5. Rank for the efficacy of six kinds of CMIs are as follows: Tanreqing (SUCRA = 0.76), Reduning (SUCRA = 0.69), Fufangkushen (SUCRA = 0.69), Danshen (SUCRA = 0.58), Xuebijing (SUCRA = 0.51), Shenmai (SUCRA = 0.20) (shown in Figure 4). The pairwise comparison (Table 4) showed that Shenmai and Fufangkushen has no statistical difference compared with the control group. There was also no significant difference among the six kinds of CMIs. Funnel plots showed that there was no publication bias and small sample effect (Supplementary Figure 3B).

**TABLE 4:** Comparison of the included CMIs in mean differences (95% CrI) for Oxygenation Index.

Control	54.02 (0.12, 106.84)	40.74 (24.08, 56.85)	9.49 (−43.6, 62.73)	57.14 (25.42, 89.26)	44.67 (7.12, 81.99)	55.05 (−5.44, 114.16)
−54.02 (−106.84, −0.12)	Reduning	−13.34 (−69.32, 42.51)	−44.36 (−119.67, 30.69)	3.24 (−57.47, 66.19)	−8.95 (−73.81, 55.61)	0.77 (−77.71, 81.79)
−40.74 (−56.85, −24.08)	13.34 (−42.51, 69.32)	Xuebijing	−31.2 (−86.97, 24.48)	16.51 (−18.62, 52.76)	4.16 (−36.53, 45)	14.15 (−47.63, 76.6)
−9.49 (−62.73, 43.6)	44.36 (−30.69, 119.67)	31.2 (−24.48, 86.97)	Shenmai	47.7 (−13.96, 110.8)	35.01 (−29.37, 99.44)	45.22 (−32.61, 125.2)
−57.14 (−89.26, −25.42)	−3.24 (−66.19, 57.47)	−16.51 (−52.76, 18.62)	−47.7 (−110.8, 13.96)	Tanreqing	−12.61 (−60.94, 36.38)	−2.29 (−70.05, 65.31)
−44.67 (−81.99, −7.12)	8.95 (−55.61, 73.81)	−4.16 (−45, 36.53)	−35.01 (−99.44, 29.37)	12.61 (−36.38, 60.94)	Danshen	10.22 (−59.26, 80.57)
−55.05 (−114.16, 5.44)	−0.77 (−81.79, 77.71)	−14.15 (−76.6, 47.63)	−45.22 (−125.2, 32.61)	2.29 (−65.31, 70.05)	−10.22 (−80.57, 59.26)	Fufangkushen

### Second Outcomes

Eight articles reported the length of ICU stay, nine articles reported Mechanical ventilation duration, eleven articles reported APACHEII score, and five articles reported Murray score. SUCRA showed that Xuebijing has the best effect on shortening the length of ICU stay (SUCRA = 0.70). Huangqi has the best effect on reducing mechanical ventilation duration (SUCRA = 0.64), APACHEII score (SUCRA = 0.96) and Murray score (SUCRA = 0.84) (Table 5). Pairwise comparison shows that in reducing the APACHEII score, Huangqi (MD = −8.77, 95% CrI: −13.3, −4.32), Danshen (MD = −5.61, 95% CrI: −9.45, −1.67), Tanreqing (MD = −3.58, 95% CrI: −6.16, −1.01) and Xuebijing (MD = −2.14, 95% CrI: −3.32, −0.87) works significantly better than that of the control group. There was also a notable difference between Huangqi and Xuebijing (MD = −6.64, 95% CrI: −11.32, −1.98) in this outcome. No statistical difference in the pairwise comparison of other outcomes.

**TABLE 5:** SUCRA for the effect of interventions on the second outcomes.

	SUCRA				
	Length of ICU stay	Mechanical ventilation duration		ApacheII score	Murray score
Control	0.22		0.21	0.00	0.22
Reduning	0.57				
Xuebijing	0.70		0.62	0.29	0.60
Shenmai			0.41		0.39
Tanreqing				0.52	0.46
Danshen				0.73	
Huangqi	0.51		0.64	0.96	0.84
Fufangkushen			0.61		

### Meta Regression and Sensitivity Analysis

In the test for heterogeneity, we found that the effects of the Oxygenation Index have a high heterogeneity. So, we try to find the source of clinical heterogeneity and methodological heterogeneity in terms of age, ALI/ARDS severity, drug combination, dose, treatment period and sample size.

First of all, we conducted meta regression for all included studies. The results showed that age, ALI/ARDS severity and sample size were not sources of heterogeneity (Supplementary Figure 4). The doses of Tanreqing, Danshen and Huangqi were the same in these studies, while Xuebijing has three different types of doses (100 ml, 150 ml, 200 ml per day). Therefore, we set dose as a covariable to conduct meta-regression for Xuebijing studies. Results showed that *p* = 0.9265, which wasn’t the source of heterogeneity. Then, we conducted a sensitivity analysis (Supplementary Figure 5). Five of the 19 studies had drug combinations, including the combination of rhubarb enema, oral administration of Lianggesan decoction, ulinastatin or high-dose ambroxol. After excluding these articles, we found that the heterogeneity is still very prominent, which indicated that drug combination was not the source of heterogeneity. Network meta-analysis after drug combination eliminated showed whether the effect or the Rank of SUCRA were not significantly different from those before the exclusion (Supplementary Table 2). Another sensitivity analysis is to remove the studies with the inconsistent treatment period. Most studies’ treatment period is seven days. Unlike most of the studies, one study of Shenmai sets the period of ten days, another Danshen study uses five days, one study of Fufangkushen did not indicate the treatment period, and one Xuebijing study picks fourteen days. The result of the Oxygenation Index in seven days would not be affected if the treatment period is longer than seven days. Therefore, we excluded the studies of five-day treatment period and unknown treatment period. There was no significant decrease in heterogeneity in the second network meta-analysis. Rank of SUCRA did not change (Supplementary Table 2), the effect of Tanreqing, Xuebijing and Shenmai didn’t change either (Supplementary Figure 5). The MD of Reduning and Danshen did not change significantly, but some differences can be found in their 95% CrI. The original 5% credible level of these two interventions was close to zero, but the value became negative after studies elimination, which made a no statistical difference between the effect of these two interventions and that of the standard treatment. This may be due to the decrease in the sample size of the two interventions after excluding the articles, which results in a wider 95% CrI. Nevertheless, since the rank and MD values have not changed, by and large, we think this network meta-analysis results of the Oxygenation Index are robust.

## Discussion

In China, as adjuvant drugs, CMIs are widely used in the treatment of various diseases, including critical diseases. The CMIs included in our research are recommended in multiple clinical guidelines of infectious diseases, Virus infection, Critically ill and so on (Tables 1). They are also commonly used for ALI/ARDS patients. While, except for Xuebijing, there is a little meta-analysis of other CMIs for ALI/ARDS treatment. Moreover, no scholar has compared their efficacy. Chen (Chen et al., 2019) made a meta-analysis of the efficacy of CMIs in the treatment of ALI/ARDS in 2019, in which showed that CMIs have great potential benefits for this disease. Yet he did not distinguish the CMIs but regards different CMIs as one intervention. Our article takes the lead in conducting a network meta-analysis of seven kinds of CMIs as adjuvant treatments in ALI/ARDS.

**FIGURE 6:**
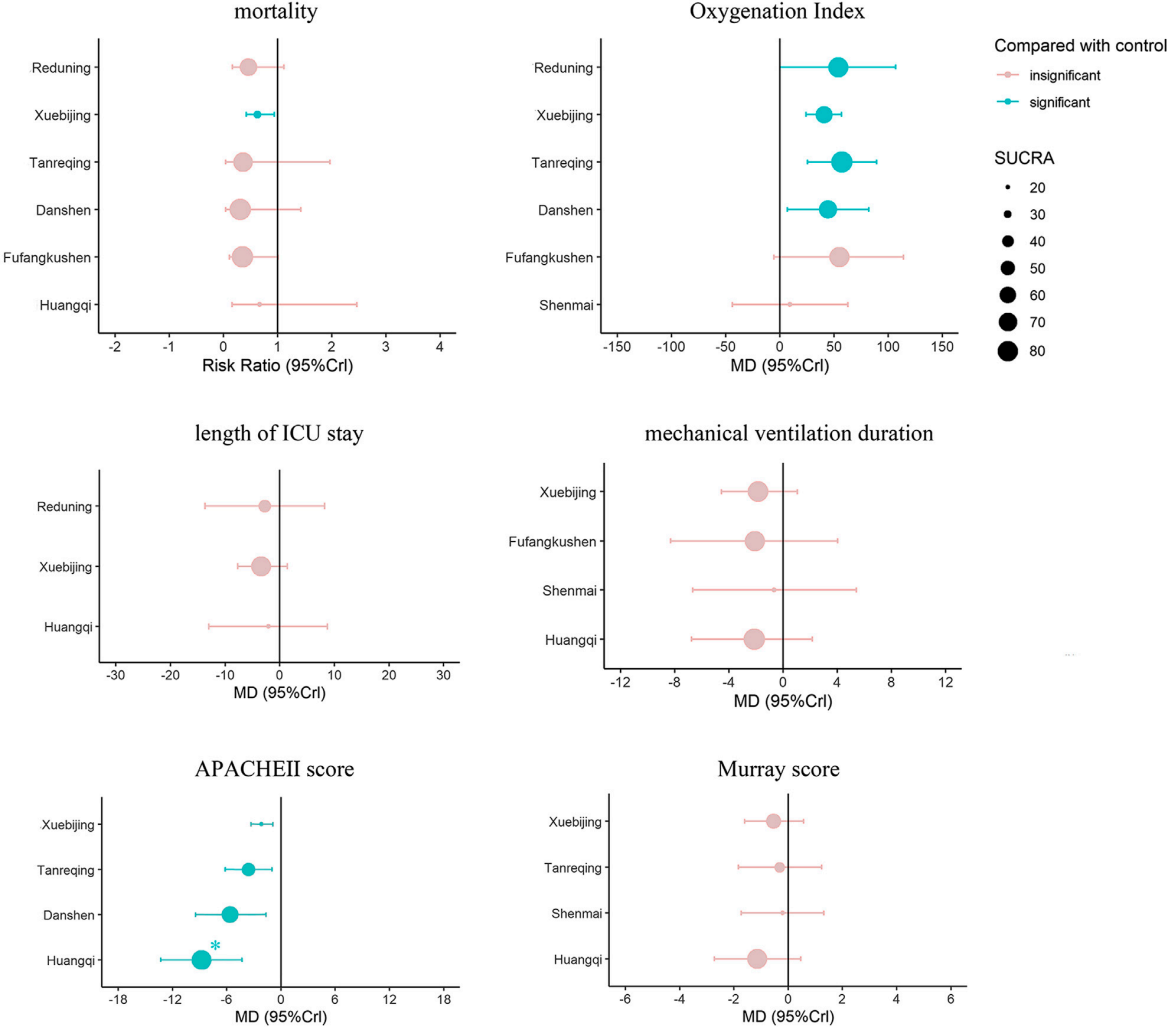
Composite forest plot of pairwise comparison and the SUCRA: The forest plot shows the RR (95% CrI) and MD (95% CrI) of each intervention compared to the control group based on Bayesian network meta-analysis. There is no significant difference between the intervention and control group if the range of 95%CrI contains the invalid line. Larger dots indicate larger SUCRA values. *: There was a statistical difference between Huangqi and Xuebijing (MD = -6.64, 95%CrI: 11.32, -1.98) in the pairwise comparison.

In order to display our results more intuitively, we present the pairwise comparison and the SUCRA of each outcome on one forest plot, using different colors to distinguish whether the results have statistical difference, and choosing different sizes of dots to represent the value of the SUCRA (shown in Figure 6).

The primary outcome of this study is mortality and Oxygenation Index, which are the most objective and critical outcomes for evaluating efficacy in ALI/ARDS. Xuebijing is the only CMI that shows a statistically different when comparing with the control group, although other five CMIs also have the tendency to reduce the mortality--their RR values are between 0.31 and 0.67 compared with the control group. This may be due to larger sample size and narrower credible interval of Xuebijing as it involved more studies. For this reason, the pairwise comparisons of each drug treatment do not show any difference either. For SUCRA, Danshen and Fufangkushen are similar and rank the top, Tanreqing and Reduning are similar, rank second, Xuebijing are close to that of Huangqi and rank last. Technically, we can't accept this result with certainty as a higher probability of ranking derives from a larger point estimate. Except for the study of Xuebijing, which has five articles referenced, all the other CMIs studies have only one relevant literature each. Therefore, the result is unrobust as the probability may change if a new study is added to the network. In fact, Xuebijing and Huangqi also showed better efficacy in other outcomes. As a consequence, we accept that using combination treatment of Xuebijing leads to lower mortality than following standard treatment without Xuebijing.

For the Oxygenation Index, our research found that it significantly improved after using Tanreqing, Reduning, Danshen or Xuebijing compare with the control group. The MD of Tanreqing and Reduning is the highest (57 and 54), while the value of Danshen and Xuebijing is relatively low (45 and 41). The 5% credible level of Reduening and Danshen is close to 0, and the number of studies is relatively few (one for Reduening and two for Danshen), so this result is not very robust. When we did sensitivity analysis, the interval estimates of these two interventions mentioned before have changed because of the exclusion of a study of Danshen. Therefore, we cannot accept the idea that they have definite benefits for the improvement of the Oxygenation Index. On the contrary, the results of Tanreqing and Xuebijing are relatively robust. In the ranking, Tanreqing is also considered to be the best CMI to improve Oxygenation Index. Tanreqing have anti-inflammatory effects and often used to treat infectious diseases in China (Wang et al., 2016). The cause of hypoxemia in ALI/ARDS is alveolar injury, and inflammation is one of the important causes of alveolar injury. The systemic inflammatory response syndrome (SIRS) triggered by the release of a large number of inflammatory mediators is also considered to be the precursor process of ALI/ARDS (Headley et al., 1997). Tanreqing can reduce the levels of IL-1, IL-6, TNF- *α* and inflammatory cytokines both in the Airway Inflammation model animal (Dong et al., 2013; Wei et al., 2016) and in patients with ALI/ARDS (Guo et al., 2018), which may be the mechanism for improving the Oxygenation Index.

In other outcomes, compared with the control group, four kinds of CMIs (Huangqi, Danshen, Tanreqing, and Xuebijing) showed significant differences in the decrease of APACHEⅡ score. After seven days of treatment using Huangqi, Danshen Tanreqing or Xuebijing, the decrease was definite. APACHE Ⅱ score includes three parts, acute physiological score (including temperature, mean arterial pressure, heart rate, respiratory rate, Oxygenation, arterial pH, Hematocrit, WBC, Serum creatinine and other serum indicators), age and chronic health evaluation. Huangqi and Danshen are herbs that can benefit qi for promoting the production of blood and activating blood circulation. In clinical, Huangqi and Danshen can influence blood pressure, heart rate, temperature and other physiological indexes. The improvement of Oxygenation and the anti-inflammatory effect triggered by Tanreqing may decline the increased heart rate, respiratory rate and WBC caused by infection. The SUCRA of Huangqi is significantly higher than that of Xuebijing, which may also benefit from the action of the above physiological indexes. APACHE II score can still reflect the severity of ICU patients and can be used as a reference for predicting mortality, as it is the most widely used disease score in ICU (Kruse et al., 1988). Therefore, Although Huangqi, Danshen and Tanreqing did not related to lower mortality, their effects on decreasing the APACHE II score also has clinical significance.

It's worth noting that, Huangqi and Danshen have only one study each, and the funnel plot is not symmetrical. Although this may be due to the heterogeneity, we cannot rule out the possibility of small sample effect and publication bias. Therefore, we should also accept this statistical result cautiously.

Huangqi and Xuebijing also ranked the highest in reducing mechanical ventilation duration and Murray score (mechanical ventilation duration: Huangqi SUCRA = 0.64, Xuebijing SUCRA = 0.62; Murray score: Huangqi SUCRA = 0.84, Xuebijing SUCRA = 0.60). Xuebijing ranked first in the length of ICU stay. However, due to the small number of studies and large credible intervals on these outcomes, there is no significant difference in the effectiveness of the CMIs and the control group. Although the clinical explanation of SUCRA is limited, Huangqi and Xuebijing have some potential to affect the above outcomes. Alveolar collapse is an important cause of refractory hypoxemia in ALI/ARDS, which may lead to lung infection and aggravate lung injury (Gattinoni et al., 2001). At present, positive end-expiratory pressure (PEEP) in mechanical ventilation is the main means to achieve the recruitment of collapsed alveoli and maintain the alveoli to remain open (Slutsky and Hudson, 2006). According to the theory of TCM, the cause of the alveolar collapse is “qi deficiency”. On this point, Huangqi has the effect of “invigorating qi”, which may act on the alveolar collapse in patients with ALI/ARDS, protect and promote the function of repairing alveolar epithelial cells, improve lung compliance, thus reduce Murray score and shorten the mechanical ventilation duration. Xuebijing has the effect of "promoting blood circulation for removing blood stasis and clearing away toxic material". Pharmacological studies have shown that it can reduce the inflammatory response, regulate immunity and blood coagulation in patients with sepsis (Kang et al., 2017). Seven hundred ten adults with severe community-acquired pneumonia from thirty-three Chinese hospitals treated with Xuebijing injection have been shown an improvement in pneumonia severity index, mortality, duration of mechanical ventilation and duration of ICU stay (Song et al., 2019). Moreover, Xuebijing can reduce the morbidity and mortality of DIC (Yin and Li, 2014), correct the abnormal blood coagulation function of DIC patients (Zheng et al., 2015). By suppressing inflammation and correcting coagulation abnormalities, the lung injury will be alleviated. As a result, the Murray score will decline, mechanical ventilation duration and ICU hospitalization time will also be reduced.

Assisting the treatment of severe diseases with traditional Chinese medicine injection has great research and application prospects. Traditional meta-analysis can provide evidence for the efficacy and safety of a single CMI, while network meta-analysis can compare the efficacy of multiple CMIs. This will provide more references for clinical medication. Furthermore, this comparison may promote the experimental research of CMIs and effective components of CMIs. Researchers can mine information from NMA research. For instance, in this study, we found that the evidence of Huangqi in reducing APACHEII score is more reliable, which may serve as a base for further studies of its mechanism. The study also shows that varieties of CMIs showed a significant effect on improving Oxygenation Index and reducing APACHEII score. Do these injections contain the same active ingredients or Chinese herbal monomers? Do these active ingredients and herbal monomers play a major role in the therapeutic effect? These questions may all become the research direction of CMI in the future. This may seem obvious, but these assumptions should be based on more reliable evidence provided by higher-quality clinical research. We believe that there will be more high-quality clinical research in the future, and NMA will become one of the main methods of TCM evidence-based medicine research.

### Limitation

This study had several limitations. First of all, the quality of the original research is not high enough. Most of the studies did not describe the allocation concealment. Besides, most studies did not adequately report the assessment of sample size, loss of follow-up and exclusion, which may further reduce the quality of evidence of the study. It is hoped that the clinical research of CMI can be more standardized in the future. Second, in terms of heterogeneity and transitivity, the factors that affect these characteristics of network meta-analysis are complex. We have done a deep exploration and model evaluation on this and faithfully presented the results. Some differences in age, ARDS severity, co-intervention, dose, and treatment period in the original study may lead to a failure of meeting the homogeneity and transitivity assumptions of network meta-analysis, resulting in a deviation in the synthesis of effect estimates. To solve this problem, we took the above characteristics as covariables to do meta-regression analysis, and through sensitivity analysis to determine the stability of statistical results. However, some covariables that cannot be analyzed, such as primary disease. The prognosis of ALI/ARDS caused by different primary diseases may be different. Unfortunately, not all studies listed the primary disease of patients. We suggest that future studies can provide more details on the primary disease. Third, for Oxygenation Index, although it is the main outcome of most ALI/ARDS studies, we should realize that the Oxygenation Index does not necessarily reflect the severity of ALI/ARDS. The increase of Oxygenation Index does not mean the reduction of mortality, as it was also influenced by PEEP (Zhu et al., 2004). Most of the studies included adopted the 1994 AECC criteria, that may affect participants included. Some patients may not conform to the diagnosis of ALI/ARDS after PEEP was set, but they may also be included in the study as the diagnostic criteria of AECC ignore the influence of PEEP (Villar et al., 2015). To solve this problem, we take the Oxygenation Index at the time of inclusion as a covariable for meta-regression, it shows that the contribution of ARDS severity to heterogeneity is not statistically significant. Thus, we believe that its influence on the result is relatively small.

## Conclusion

This network meta-analysis shows that a combination of the standard treatment and Xuebijing is associated with lower mortality compared with the standard scheme. The efficacy of other CMIs is uncertain. More credible evidence indicates that Tanreqing and Xuebijing have the best effect on improving the Oxygenation Index. Four kinds of CMIs (Huangqi, Danshen, Tanreqing and Xuebijing) can significantly reduce APACHE II scoreHuangqi works better than Xuebijing in pairwise comparison. Huangqi and Xuebijing ranked the highest in reducing mechanical ventilation duration and Murray score, while Xuebijing ranked first on shortening the length of ICU stay (but they are not statistically significant). To sum up, the efficacies of Xuebijing, Tanreqing and Huangqi have varying effect on different outcomes, as adjuvant drugs of ALI/ARDS, but more evidence is needed.

## Data Availability

The original contributions presented in the study are included in the article/Supplementary material, further inquiries can be directed to the corresponding authors.

## Abbreviations

CMI, chinese medicine injection; ALI, acute lung injury; ARDS, acute respiratory distress syndrome; CNKI, China national knowledge infrastructure; RCTs, randomized clinical trials; AECC, American-European consensus conference; RR, risk ratios; MD, mean difference; 95% CrI: 95% credible intervals; DIC, (1) deviance information criterion, (2) disseminated intravascular coagulation; PSRF, potential scale reduction parameter; SUCRA, surface under cumulative ranking; SIRS, systemic inflammatory response syndrome; IL, interleukin; TNF, tumor necrosis factor; WBC, white blood cell; PEEP, positive end-expiratory pressure.
