# Supplementary material for: Comparative Efficacy of Seven Kinds of Chinese Medicine Injections in Acute Lung Injury and Acute Respiratory Distress Syndrome: A Network Meta-analysis of Randomized Controlled Trials
Source: Front Pharmacol. 2021 Mar 9;12:627751. doi: 10.3389/fphar.2021.627751 (PMC7985440; doi:10.3389/fphar.2021.627751)
Supplement: Supplementary file 1 [file datasheet1.docx]

Supplementary Material

# Supplementary Data

## Search strategy

The Cochrane Library

#1 Medicine, Chinese Traditional[MeSH Terms] OR "Traditional Chinese Medicine" OR "Chung I Hsueh" OR "Zhong Yi Xue" OR "Chinese Traditional Medicine" OR "Chinese Medicine" OR Chinese herb*

#2xuebijing OR tanreqing OR reduning OR xiyanping OR qingkailing OR shengmai OR shenmai OR zhenqifuzheng OR shenqifuzheng OR chuanxiongqin OR shenfu OR danshen OR yanhuning

#3 injections[MeSH Terms] OR Inject*

#4 Respiratory Distress Syndrome, Adult[MeSH Terms] OR "Respiratory Distress Syndrome" OR "Shock Lung" OR ARDS OR ARDSs

#5 acute lung injury[MeSH Terms] OR "acute lung injury" OR ALI

#6 "acute respiratory failure" OR ARF

#7 #1 OR #2

#8 #7 AND #3

#9 #4 OR #5 OR #6

#10 #8 AND #9

PUBMED

Search ((((((randomized controlled trial[MeSH Terms]) OR “randomized controlled trial”) OR “controlled clinical trial”) OR random*) OR trial)) AND ((((((((((respiratory distress syndrome, adult[MeSH Terms]) OR "Respiratory Distress Syndrome") OR "Shock Lung") OR ARDS) OR ARDSs)) OR (((acute lung injury[MeSH Terms]) OR "acute lung injury") OR ALI)) OR (("acute respiratory failure") OR ARF))) AND (((((((((((((((((xuebijing) OR tanreqing) OR reduning) OR xiyanping) OR qingkailing) OR yanhuning) OR shenmai) OR shengmai) OR zhenqifuzheng) OR shenqifuzheng) OR chuanxiongqin) OR shenfu) OR danshen)) OR (((((((medicine, chinese traditional[MeSH Terms]) OR "Traditional Chinese Medicine") OR "Chung I Hsueh") OR "Zhong Yi Xue") OR "Chinese Traditional Medicine") OR "Chinese Medicine") OR Chinese herb*))) AND ((injections[MeSH Terms]) OR Inject*)))

- 1. **References of clinical guidelines**

Association, E.P.B.o.t.C.P. (2015). Expert consensus on the diagnosis and treatment of acute paraquat intoxication (2013). Chin J Crit Care Med 33(6), 484-489.

Chen, K., Wu, Z., Zhu, M., Mao, J., and Xu, H. (2016). The expert consensus on the combination of Chinese and Western medicine for the treatment of chronic heart failure. Chinese Journal of Integrated Traditional and Western Medicine 36(02), 133-141.

Chen, K., Zhang, M., and Huo, Y. (2014). The expert consensus on the combination of Chinese and Western medicine for the treatment of acute myocardial infarction. Chinese Journal of Integrated Traditional and Western Medicine 34(04), 389-395.

Chinese Research Hospital Association of Critical Care Medicine, and Youth Committee of Chinese Research Hospital Association of Critical Care Medicine (2020). The expert consensus on the diagnosis and treatment of severe and critical COVID-19 (revision). Chinese Critical Care Medicine (03), 269-274.

Ding, Y., Sun, X., Bi, L., and Zhang, X. (2011). Guidelines on diagnosis and treatment of allergic cyanosis. Journal of Pediatrics of Traditional Chinese Medicine 7(06), 1-4.

Fang, B., Li, Z., Li, Y., and Wang, G. (2018). The expert consensus on the combination of Chinese and Western medicine for the treatment of acute ischemic stroke. Chinese Critical Care Medicine 30(03), 193-197.

Gao, L. (2018). The expert consensus on the combination of Chinese and Western medicine for the treatment of chronic cerebral ischemia. Chinese Journal of Integrated Traditional and Western Medicine 38(10), 1161-1167.

Li, J., and Yu, X. (2014). Chinese medicine guidelines for chronic pulmonary heart disease(2014). Journal of Traditional Chinese Medicine 55(06), 526-531.

Li, Z. (2014). The expert consensus on the combination of Chinese and Western medicine for the treatment of multiple organ dysfunction syndrome in the elderly (draft). Chinese Critical Care Medicine 26(07), 449-453.

National Health and Family Planning Commission (2017). Guidelines on diagnosis and treatment of human infection with H7N9 avian influenza (H7N9) virus (2017 version). Chin J Viral Dis 7(1), 1-4.

National Health Commisson of the People's Republic of China, and National Administration of Traditional Chinese Medicine (2020). Diagnosis and treatment program of COVID-19 (Trial version 8). China Medicine 15(10), 1494-1499.

Professional Committee on Infectious Diseases, C.a.W.M.A. (2019). The expert consensus on the combination of Chinese and Western medicine for the treatment of severe fever with thrombocytopenia syndrome. Global Traditional Chinese Medicine 12(10), 1506-1511.

Sub-Health Professional Committee of China Healthcare International Exchange Promotion Association (2020). The expert consensus on the combination of Chinese and Western medicine for the treatment of asthmatic bronchitis. Research of Integrated Traditional Chinese and Western Medicine 12(01), 32-35.

Wang, L., and Chai, Y. (2017). Emergency expert consensus on sepsis complicated with disseminated intravascular coagulation. Chinese Journal of Clinical Pathologist 9(03), 129-132.

Wang, X., Shao, Z., and Ba, Y. (2011). Guidelines on diagnosis and treatment of chronic glomerulonephritis. Chinese Medicine Modern Distance Education of China 9(09), 129-132.

Wang, X., Yu, J., and Jin, S. (2020). The expert consensus on the combination of Chinese and Western medicine for the treatment of sepsis complicated with ALI. Chinese Journal of Surgery of Integrated Traditional and Western Medicine 26(03), 400-408.

Wang, Y., Chen, Y., Lv, C., and et.al. (2019). Chinese expert consensus on the diagnosis and treatment of stroke -associated pneumonia. Chinese Journal of Stroke 14(12), 1251-1262.

Wang, Z., Wei, J., Zhu, H., Cao, Y., Yu, X., Chen, Y., et al. (2020). Emergency expert consensus on early prevention and blocking of sepsis in China. Journal of Clinical Emergency 21(07), 517-529.

Yu, X., Xue, Y., and Li, J. (2019). Guidelines on diagnosis and treatment of Chinese medicine for community acquired pneumonia (2018 revision). Journal of Traditional Chinese Medicine 60(04), 350-360.

Zhang, S., Li, Q., Li, H., Wang, H., and Tang, Y. (2013). The expert consensus on the diagnosis and treatment of acute pancreatitis in Chinese medicine. China Journal of Traditional Chinese Medicine and Pharmacy 28(06), 1826-1831.

# Supplementary Figures and Tables

## Supplementary Figures


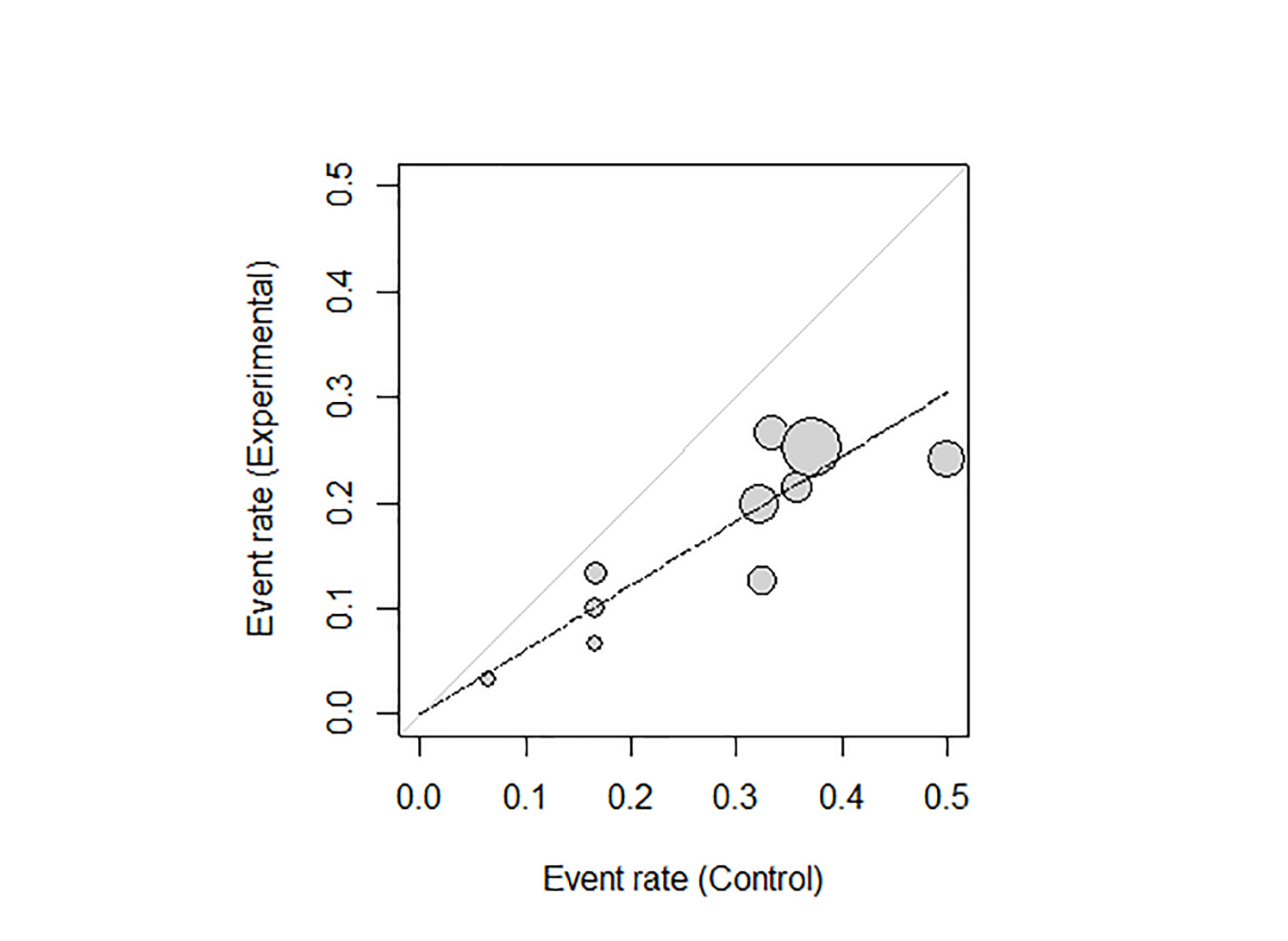


**Supplementary Figure 1.** L'Abbe diagram of mortality

**
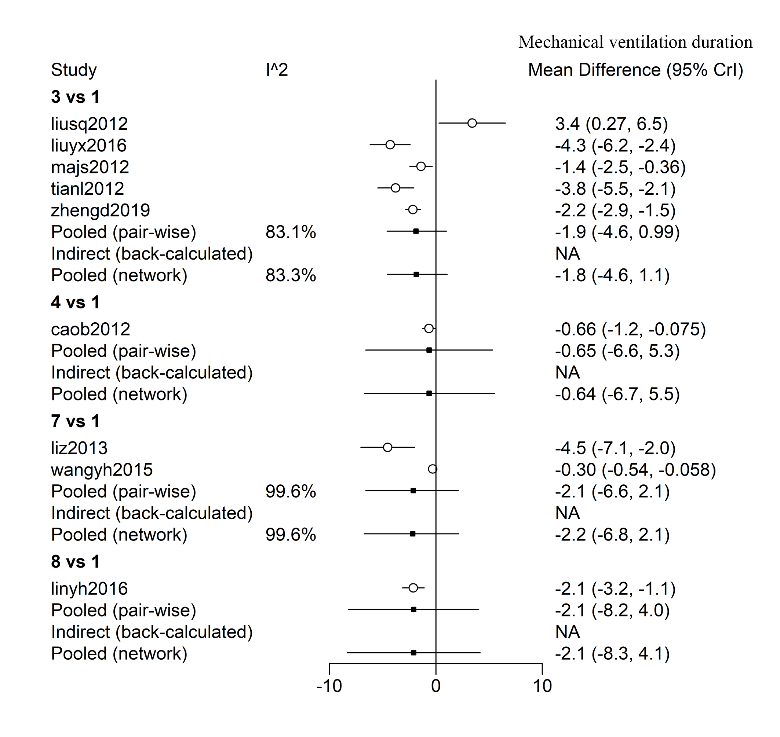

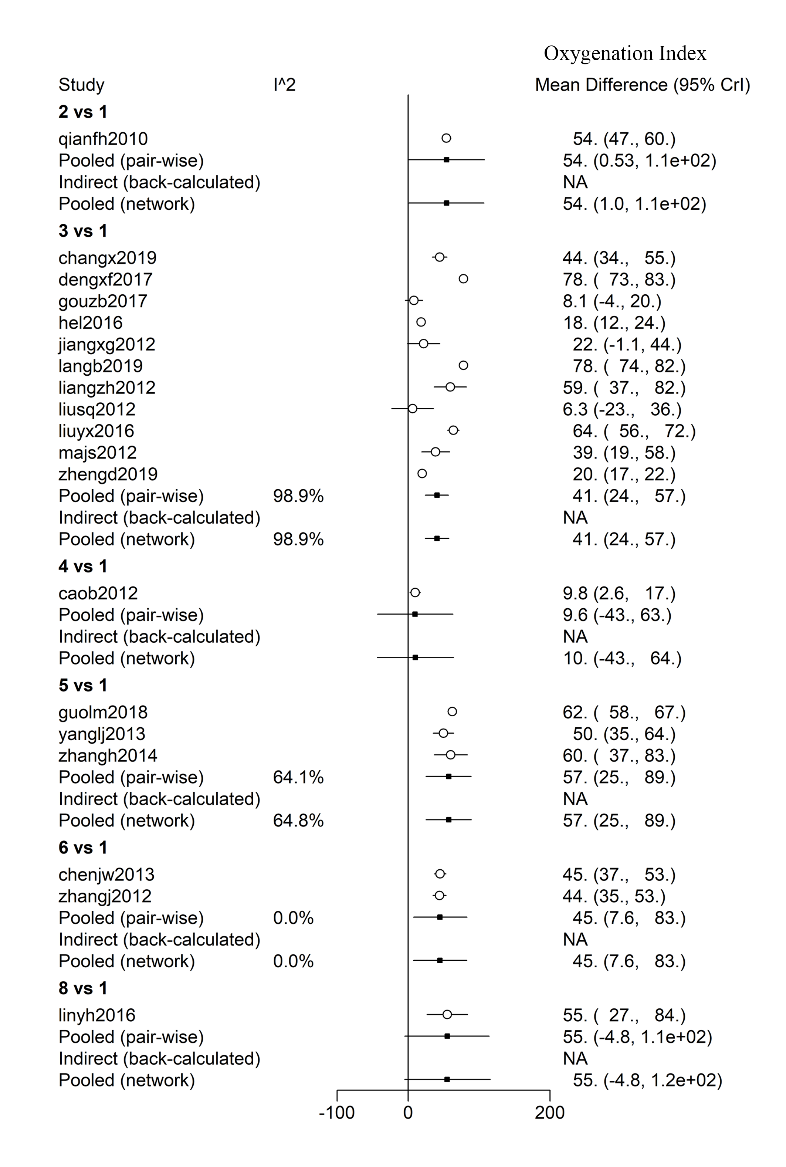

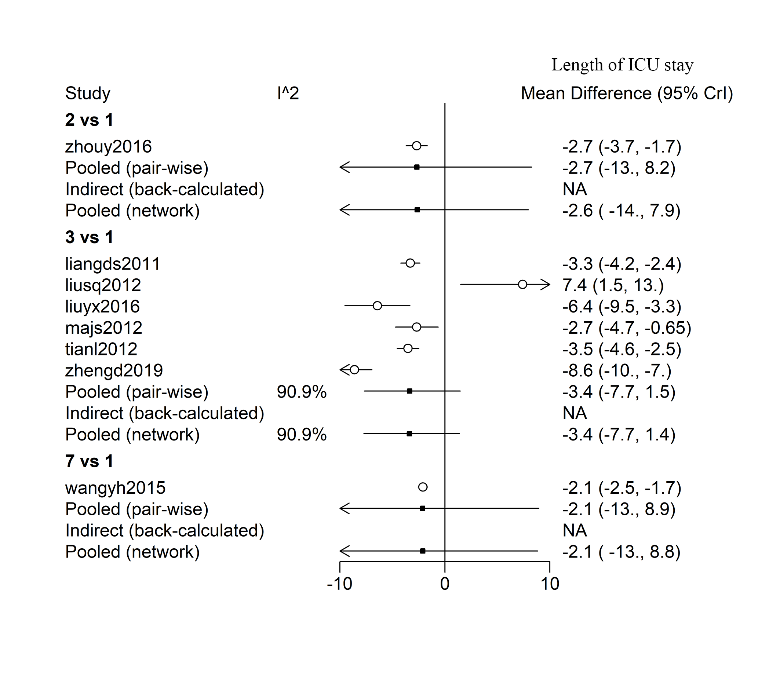
(B)**

**
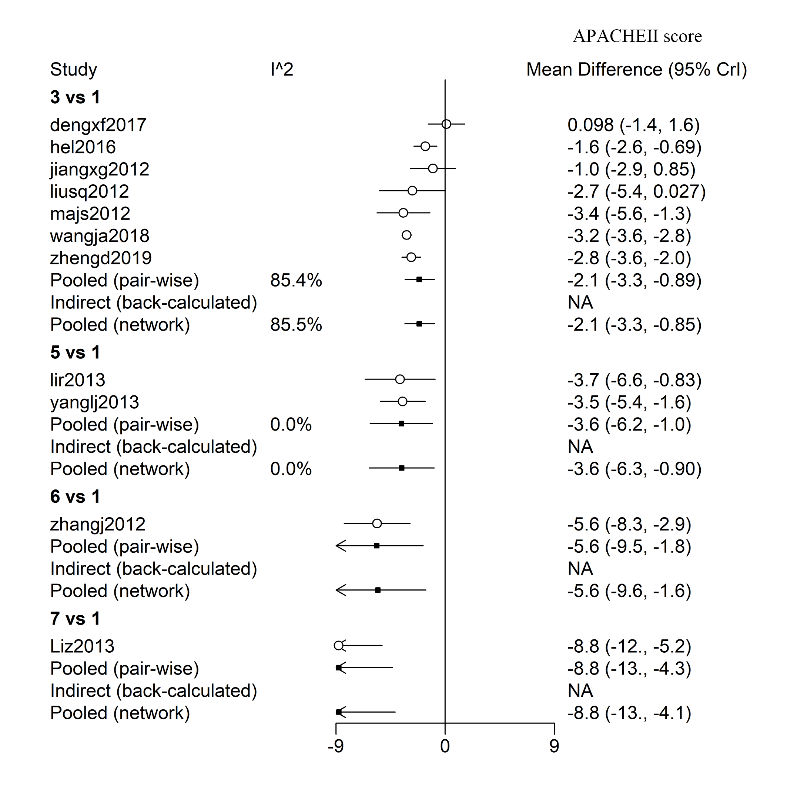
(A)**

**
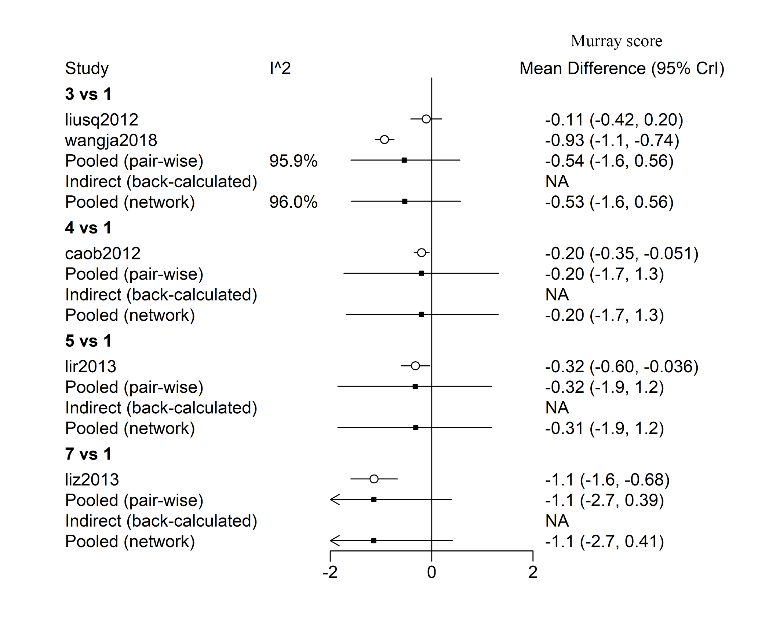
(C)**

**(E) (D)**

**Supplementary Figure 2.** Forest Plots of Direct, Indirect, and Pooled Comparisons: (A) Oxygenation Index; (B) Length of ICU stay; (C) Mechanical ventilation duration; (D) APACHEⅡ score; (E) Murray score

**
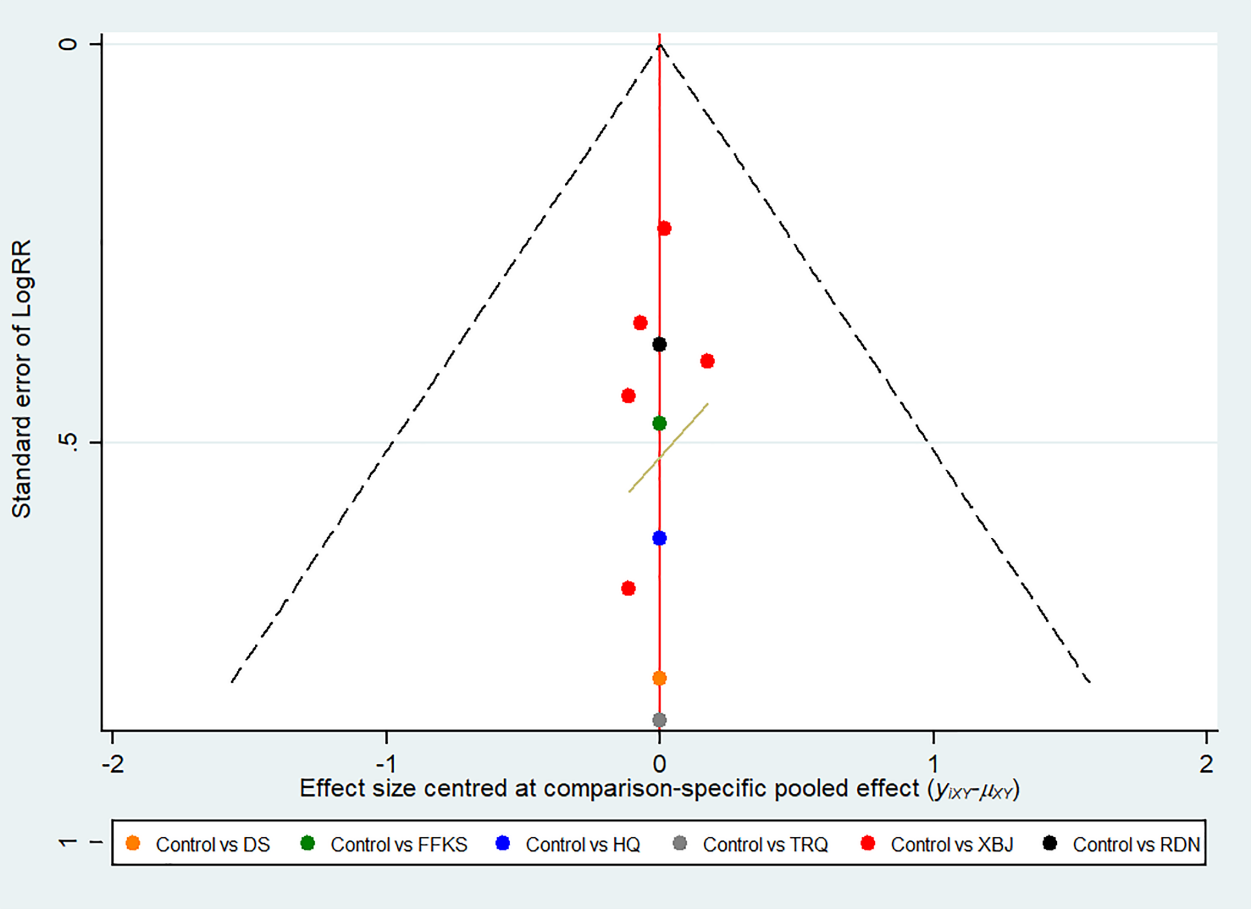
**

**(A)**

**
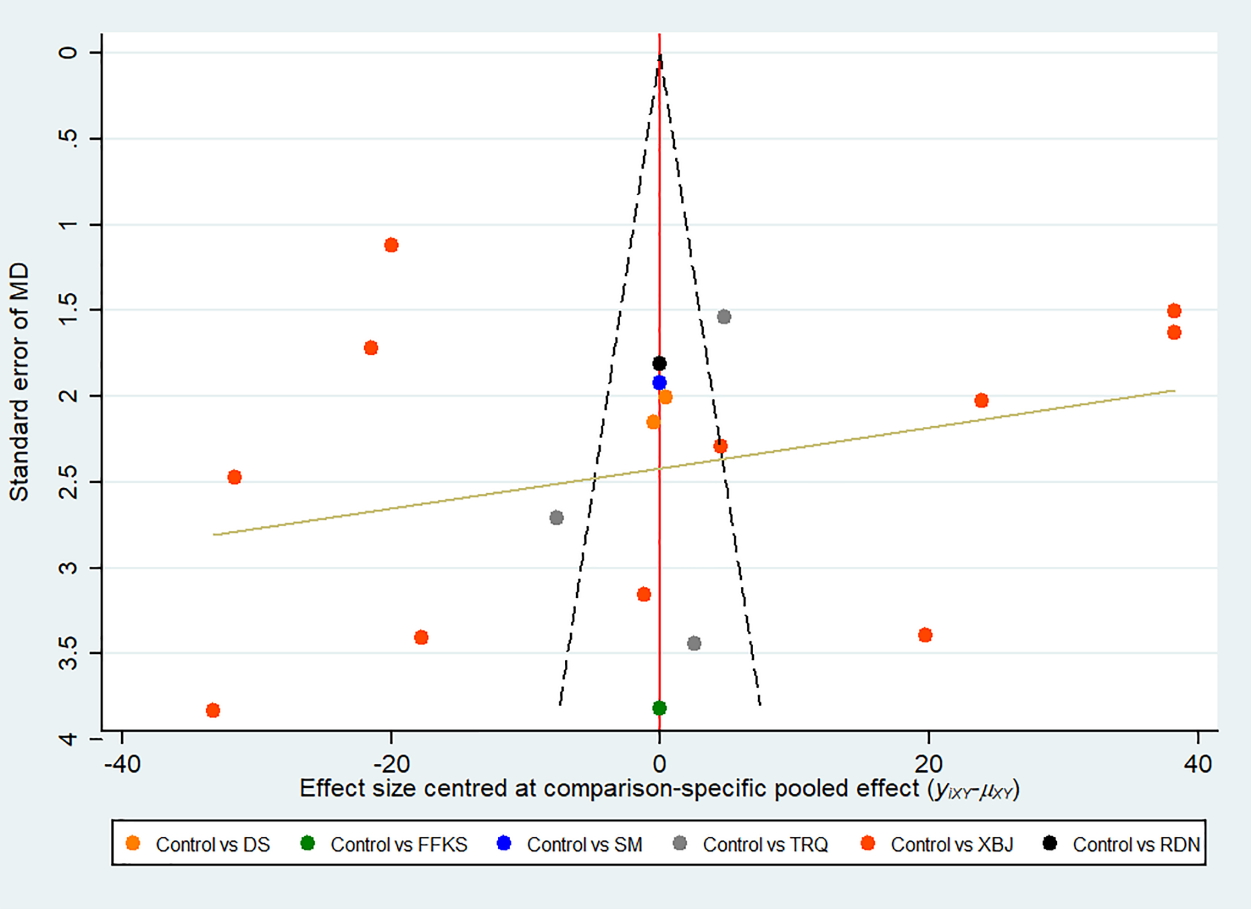
**

**
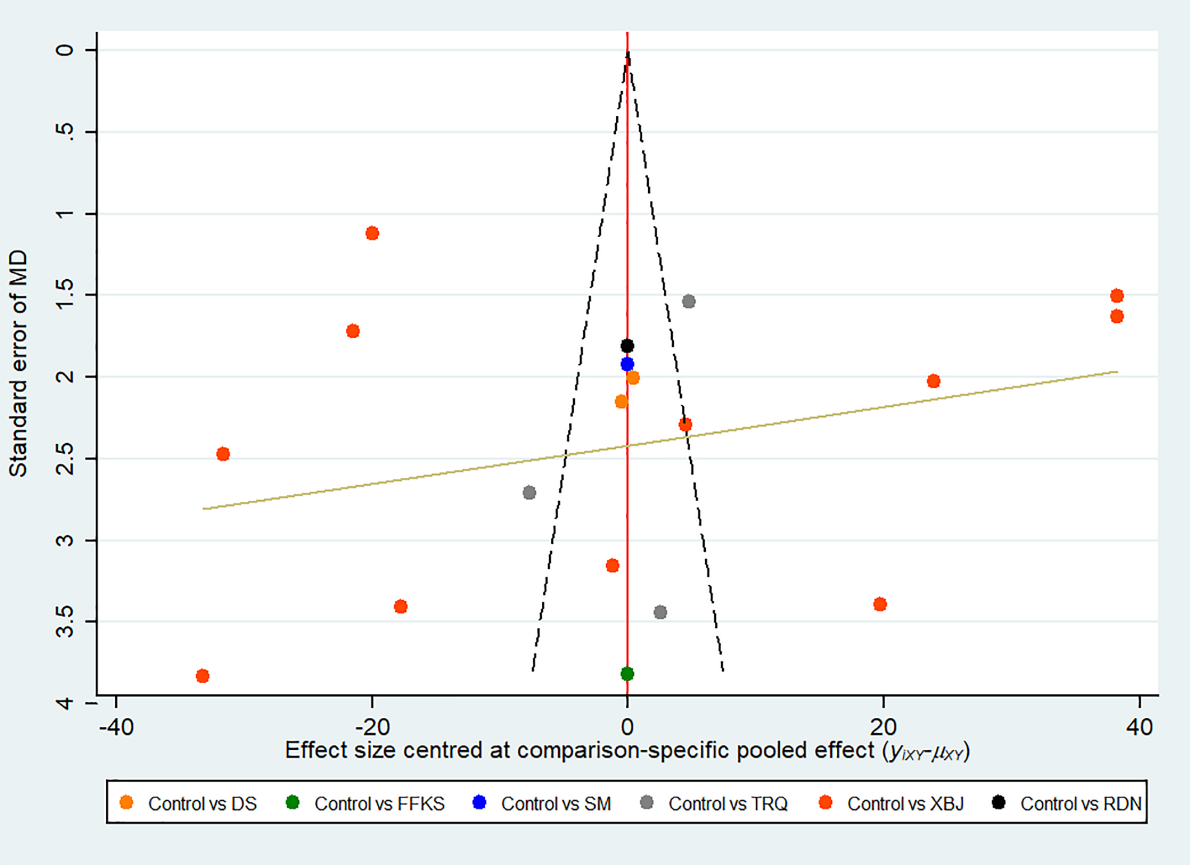
(B)**

**
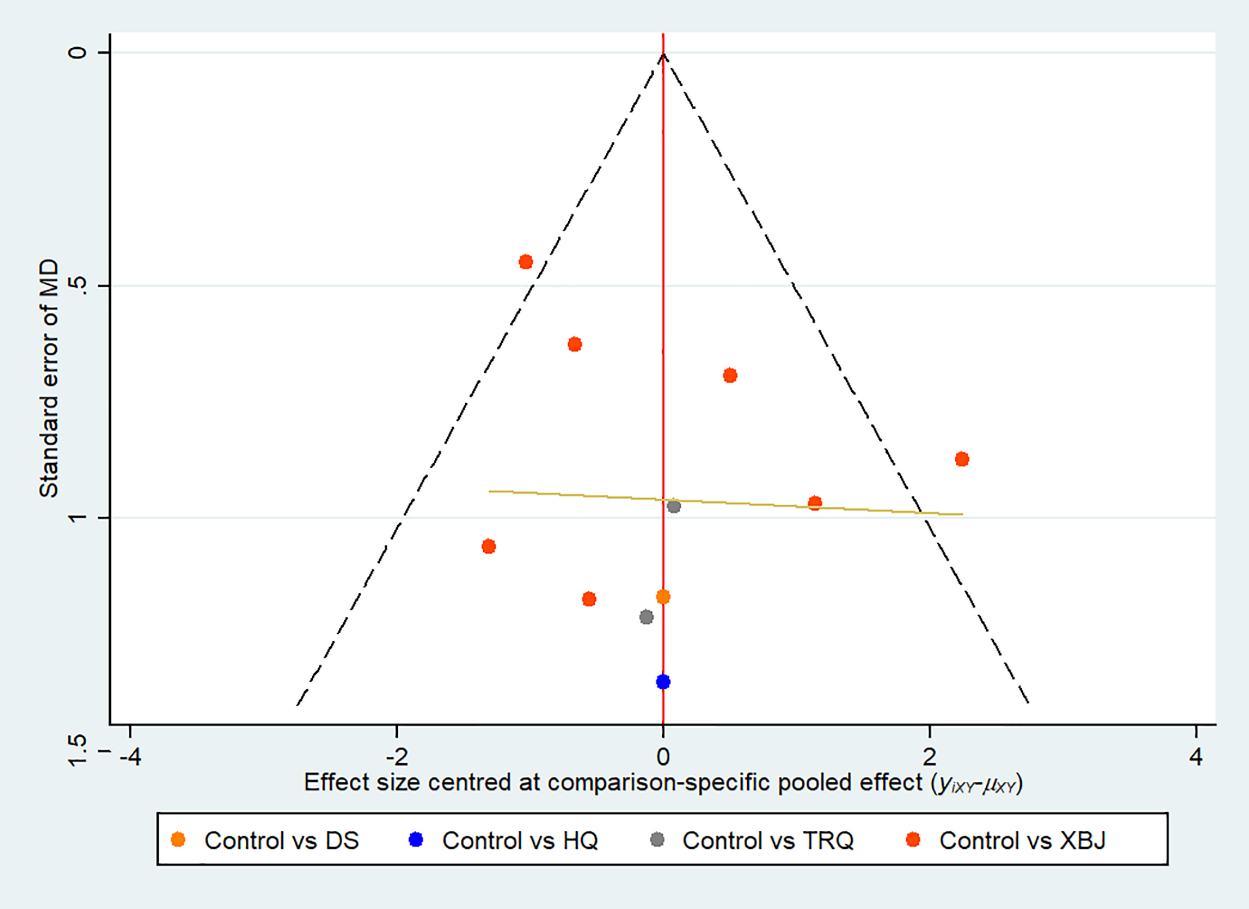
**

**(C)**

**Supplementary Figure 3.** Funnel Plots for mortality (A), Oxygenation index (B) and APACHEⅡ score (C)


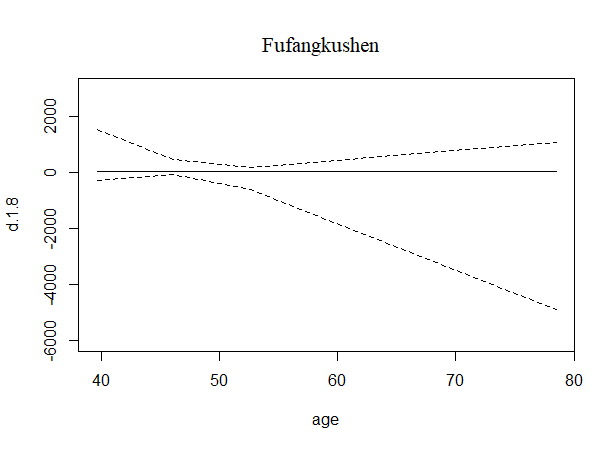

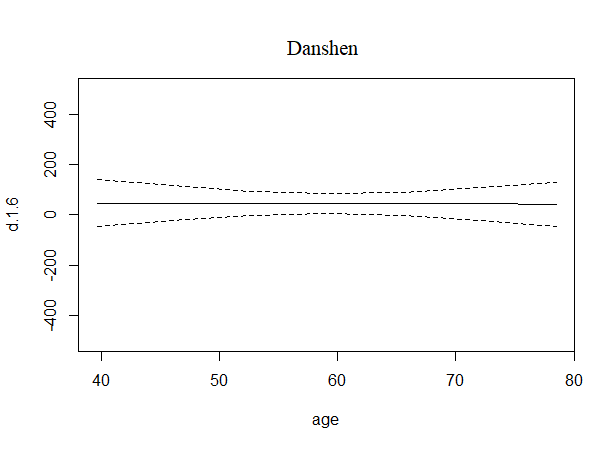

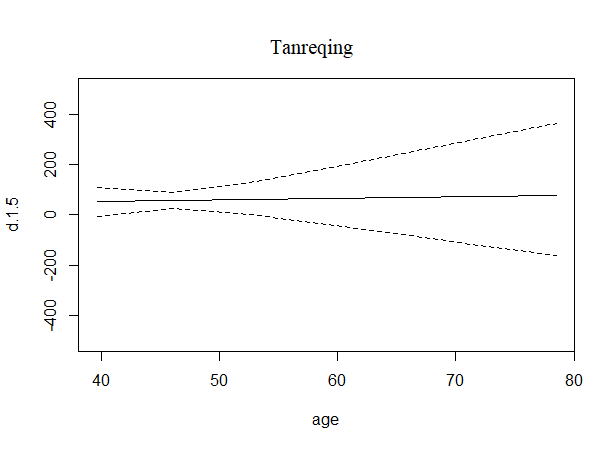

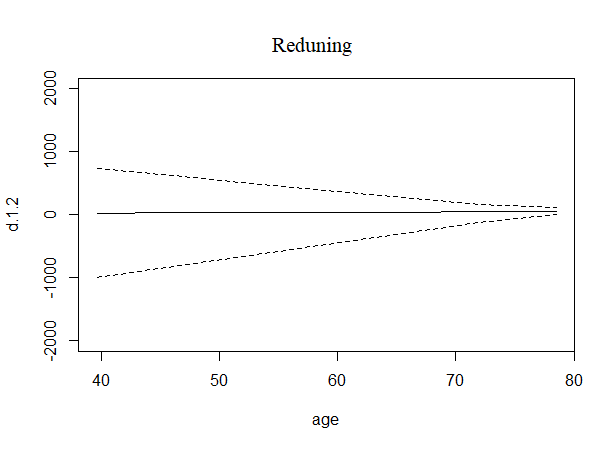

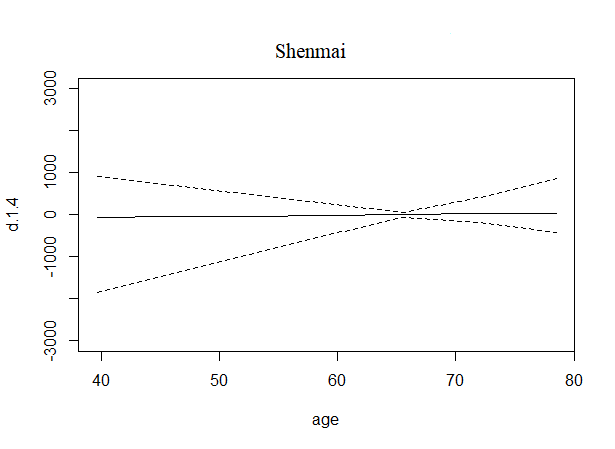

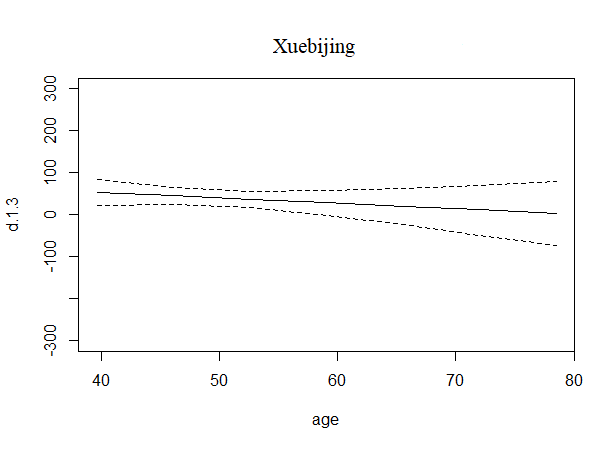


**
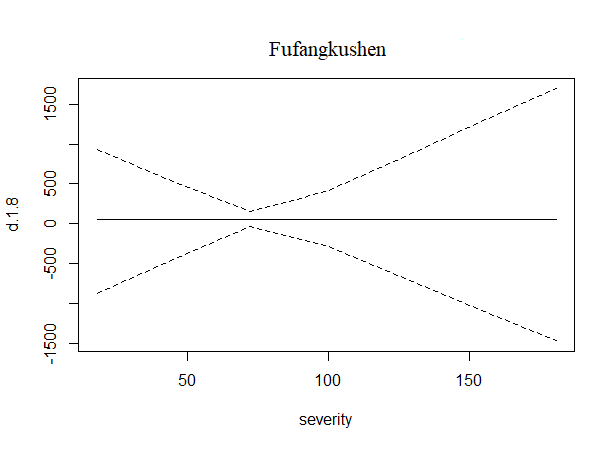

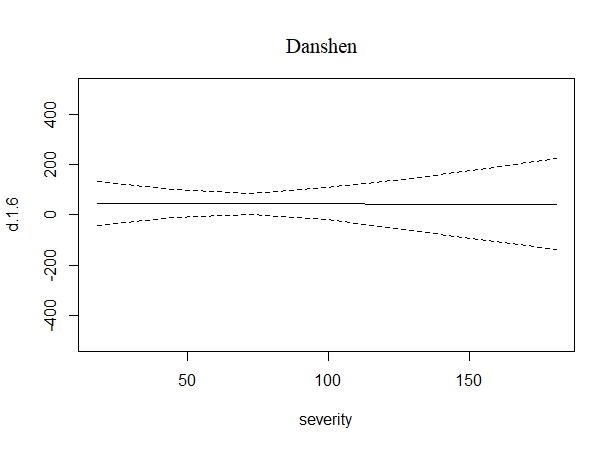

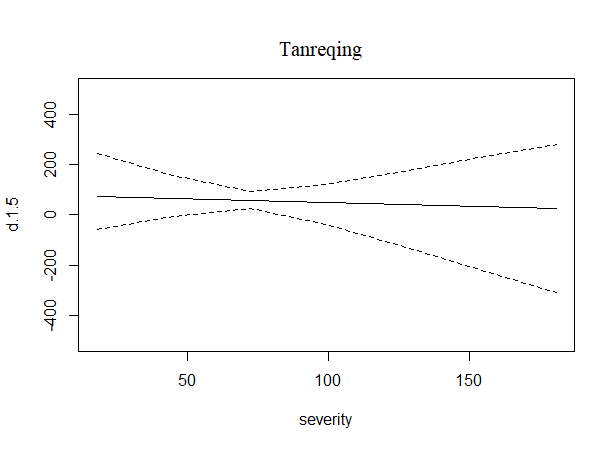

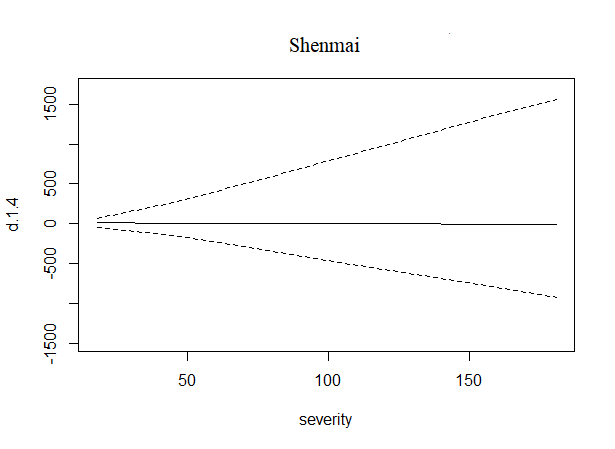

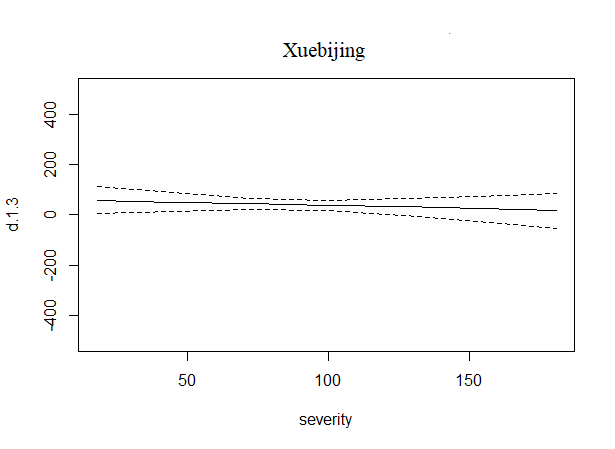

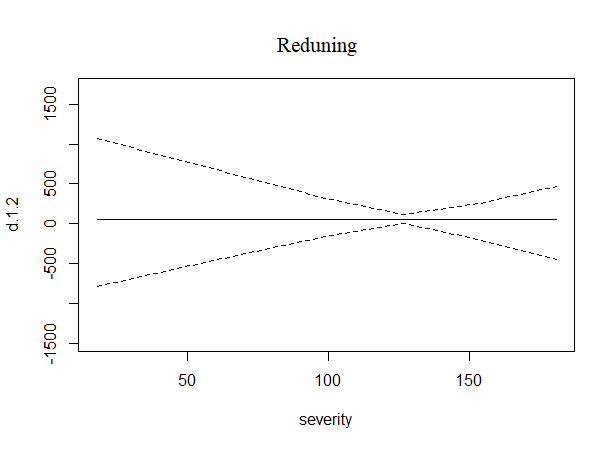
(A)**

**(B)**

**
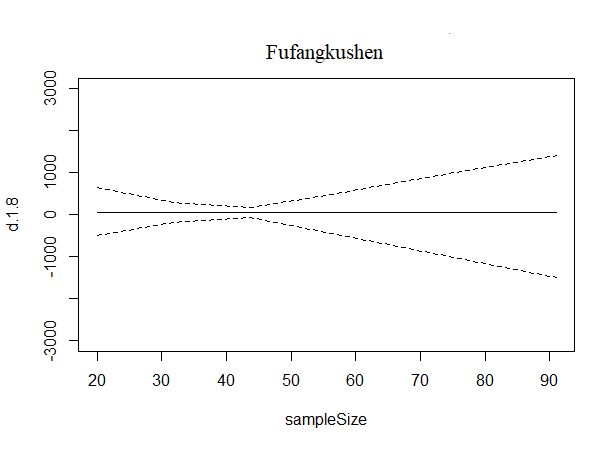

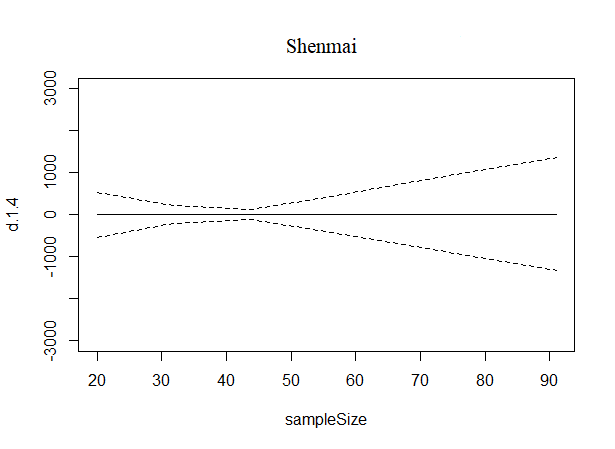

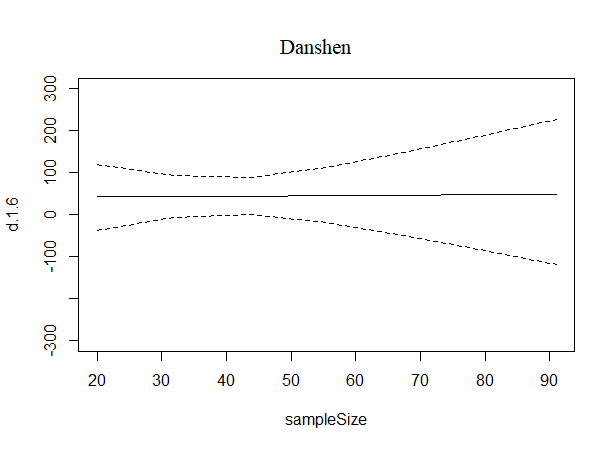

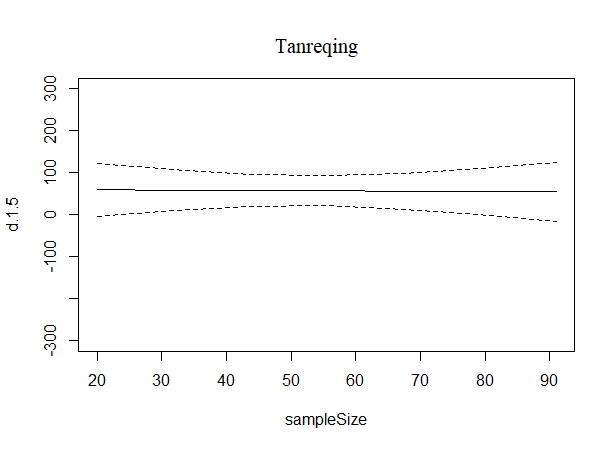

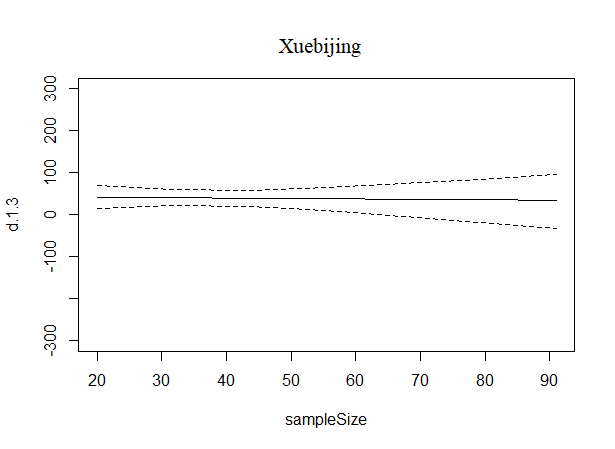

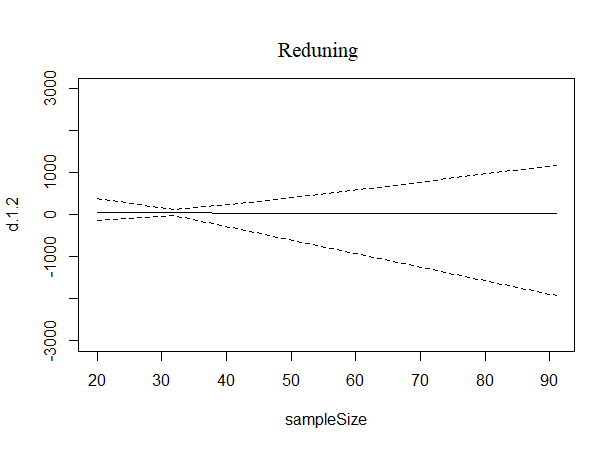
(C)**

**Supplementary Figure 4.** Results of Network Metaregression for Age, ARDS Severity and Samplesize: (A) Treatment effect of each intervention versus covariate (Age): control (Reference); (B) Treatment effect of each intervention versus covariate (ARDS severity): control (Reference); (C) Treatment effect of each intervention versus covariate (Samplesize): control (Reference).


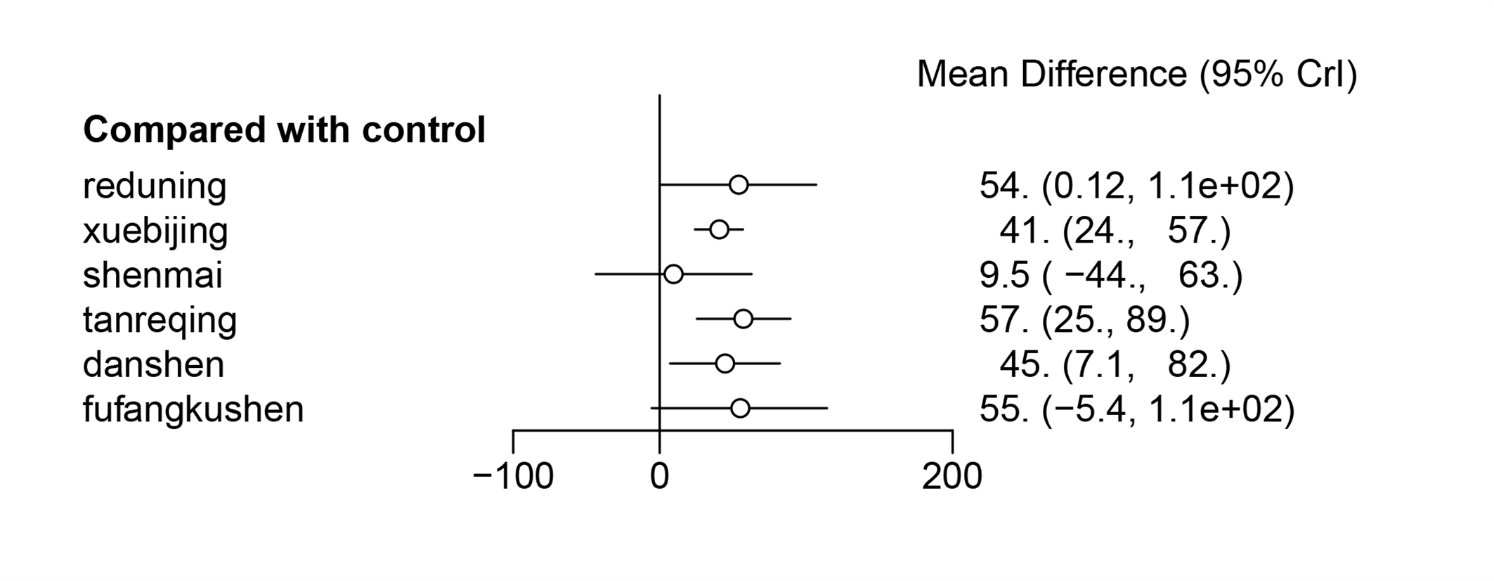


**(A)**

**
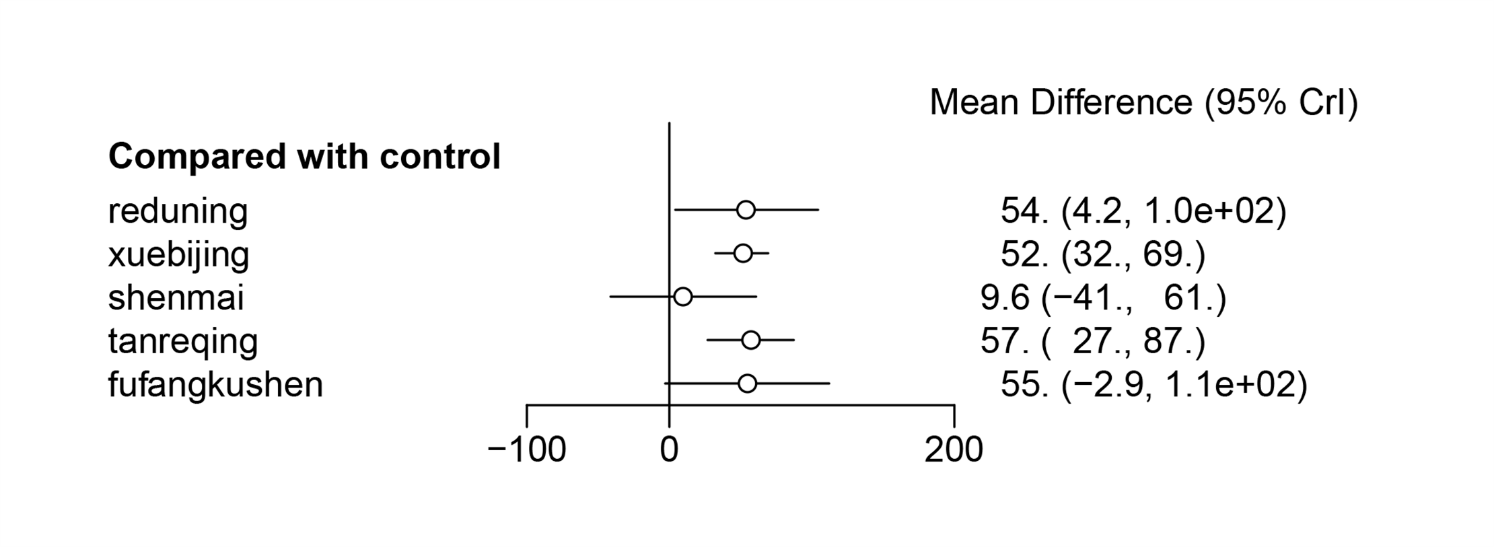
**

**(B)**

**
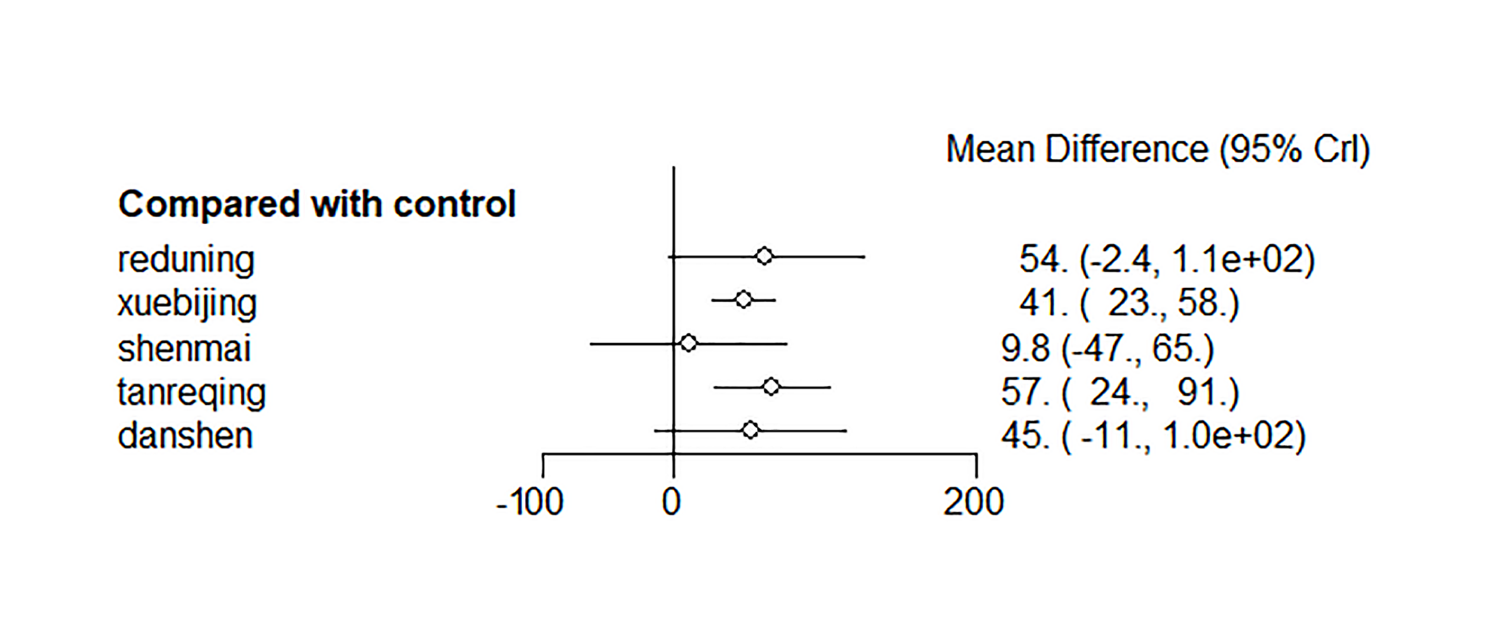
**

**(C)**

**Supplementary Figure 5.** Effect in sensitivity analysis for Oxygenation Index: (A) All included studies; (B) Drug combination eliminated; (C) Different treatment period eliminated

## Supplementary Tables

**Supplementary Tables 1.** Assessment of Model Fits for Outcomes

|  | Dbar | data points | pD | DIC | I^2^ |
| --- | --- | --- | --- | --- | --- |
| Fixed effects model | 16.37625 | 20 | 15.51859 | 31.89483 | 0% |
| Random effects model | 17.20828 | 20 | 16.49139 | 33.69967 | 0% |

a. mortality: Based on the above information, we chose Random effect model. Potential scale reduction factors of this model is 1. the convergence is good.

|  | Dbar | data points | pD | DIC | I2 |
| --- | --- | --- | --- | --- | --- |
| Fixed effects model | 895.91653 | 38 | 25.05642 | 920.97295 | 96% |
| Random effects model | 37.77664 | 38 | 36.97551 | 74.75215 | 2% |

b. Oxygenation index: Based on the above information, Random effect model is the preferred model. Potential scale reduction factors of this model is 1. the convergence is good.

|  | Dbar | data points | pD | DIC | I2 |
| --- | --- | --- | --- | --- | --- |
| Fixed effects model | 56.66510 | 14 | 10.01684 | 66.68194 | 77% |
| Random effects model | 14.84986 | 14 | 13.89782 | 28.74767 | 12% |

c. length of ICU stay: Based on the above information, Random effect model is the preferred model. Potential scale reduction factors of this model is 1. the convergence is good.

|  | Dbar | data points | pD | DIC | I^2^ |
| --- | --- | --- | --- | --- | --- |
| Fixed effects model | 46.71226 | 18 | 13.13388 | 59.84614 | 64% |
| Random effects model | 18.86329 | 18 | 17.73926 | 36.60255 | 10% |

d. mechanical ventilation duration: Based on the above information, Random effect model is the preferred model. Potential scale reduction factors of this model is 1. the convergence is good.

|  | Dbar | data points | pD | DIC | I^2^ |
| --- | --- | --- | --- | --- | --- |
| Fixed effects model | 42.46073 | 22 | 15.00436 | 57.46509 | 51% |
| Random effects model | 21.72704 | 22 | 20.06722 | 41.79426 | 3% |

e. APACHEⅡ score: Based on the above information, Random effect model is the preferred model. Potential scale reduction factors of this model is 1. the convergence is good.

|  | Dbar | data points | pD | DIC | I^2^ |
| --- | --- | --- | --- | --- | --- |
| Fixed effects model | 29.195858 | 10 | 8.998494 | 38.194351 | 69% |
| Random effects model | 10.09078 | 10 | 10.02484 | 20.11562 | 11% |

f. Murray score: Based on the above information, Random effect model is the preferred model. Potential scale reduction factors of this model is 1. the convergence is good.

**Supplementary Tables 2.** Rank of SUCRA for Oxygenation index in sensitivity analysis

|  | A | | B | | C | |
| --- | --- | --- | --- | --- | --- | --- |
|  | SUCRA | RANK | SUCRA | RANK | SUCRA | RANK |
| Control | 0.07 | 6 | 0.08 | 6 | 0.09 | 6 |
| Reduning | 0.69 | 2 | 0.67 | 3 | 0.71 | 2 |
| Xuebijing | 0.51 | 4 | 0.65 | 4 | 0.56 | 4 |
| Shenmai | 0.20 | 5 | 0.19 | 5 | 0.23 | 5 |
| Tanreqing | 0.76 | 1 | 0.73 | 1 | 0.79 | 1 |
| Danshen | 0.58 | 3 |  |  | 0.62 | 3 |
| Fufangkushen | 0.69 | 2 | 0.68 | 2 |  |  |

A. All included studies; B. Drug combination eliminated; C. Different treatment period eliminated
